# *RNF213* Variants, Vasospastic Angina, and Risk of Fatal Myocardial Infarction

**DOI:** 10.1001/jamacardio.2024.1483

**Published:** 2024-06-18

**Authors:** Keiko Hikino, Satoshi Koyama, Kaoru Ito, Yoshinao Koike, Masaru Koido, Takayoshi Matsumura, Ryo Kurosawa, Kohei Tomizuka, Shuji Ito, Xiaoxi Liu, Yuki Ishikawa, Yukihide Momozawa, Takayuki Morisaki, Yoichiro Kamatani, Taisei Mushiroda, Chikashi Terao

**Affiliations:** 1 Laboratory for Pharmacogenomics, RIKEN Center for Integrative Medical Sciences, Yokohama City, Japan; 2 Laboratory for Cardiovascular Genomics and Informatics, RIKEN Center for Integrative Medical Sciences, Yokohama City, Japan; 3 Laboratory for Statistical and Translational Genetics, RIKEN Center for Integrative Medical Sciences, Yokohama City, Japan; 4 Department of Orthopedic Surgery, Hokkaido University Graduate School of Medicine, Sapporo, Japan; 5 Division of Molecular Pathology, Institute of Medical Science, The University of Tokyo, Tokyo, Japan; 6 Laboratory of Complex Trait Genomics, Department of Computational Biology and Medical Sciences, Graduate School of Frontier Sciences, The University of Tokyo, Tokyo, Japan; 7 Division of Human Genetics, Center for Molecular Medicine, Jichi Medical University, Shimotsuke, Japan; 8 Department of Orthopedic Surgery, Shimane University Faculty of Medicine, Izumo, Japan; 9 Laboratory for Genotyping Development, RIKEN Center for Integrative Medical Sciences, Yokohama City, Japan; 10 Clinical Research Center, Shizuoka General Hospital, Shizuoka, Japan; 11 Department of Applied Genetics, The School of Pharmaceutical Sciences, University of Shizuoka, Shizuoka, Japan

## Abstract

**Importance:**

Vasospastic angina (VSA) is vasospasm of the coronary artery and is particularly prevalent in East Asian populations. However, the specific genetic architecture for VSA at genome-wide levels is not fully understood.

**Objective:**

To identify genetic factors associated with VSA.

**Design, Setting, and Participants:**

This was a case-control genome-wide association study of VSA. Data from Biobank Japan (BBJ; enrolled patients from 2002-2008 and 2013-2018) were used, and controls without coronary artery disease (CAD) were enrolled. Patients from the BBJ were genotyped using arrays or a set of arrays. Patients recruited between 2002 and 2005 were classified within the first dataset, and those recruited between 2006 and 2008 were classified within the second dataset. To replicate the genome-wide association study in the first and second datasets, VSA cases and control samples from the latest patients in the BBJ recruited between 2013 and 2018 were analyzed in a third dataset.

**Exposures:**

Single-nucleotide variants associated with VSA.

**Main Outcomes and Measures:**

Cases with VSA and controls without CAD.

**Results:**

A total of 5720 cases (mean [SD] age, 67 [10] years; 3672 male [64.2%]) and 153 864 controls (mean [SD] age, 62 [15] years; 77 362 male [50.3%]) in 3 datasets were included in this study. The variants at the *RNF213* locus showed the strongest association with VSA across the 3 datasets (odds ratio [OR], 2.34; 95% CI, 1.99-2.74; *P* = 4.4 × 10^−25^). Additionally, rs112735431, an Asian-specific rare deleterious variant (p.Arg4810Lys) experimentally shown to be associated with reduced angiogenesis and a well-known causal risk for Moyamoya disease was the most promising candidate for a causal variant explaining the association. The effect size of rs112735431 on VSA was distinct from that of other CADs. Furthermore, homozygous carriers of rs112735431 showed an association with VSA characterized by a large effect estimate (OR, 18.34; 95% CI, 5.15-65.22; *P* = 7.0 × 10^−6^), deviating from the additive model (OR, 4.35; 95% CI, 1.18-16.05; *P* = .03). Stratified analyses revealed that rs112735431 exhibited a stronger association in males (χ^2^_1_ = 7.24; *P* = .007) and a younger age group (OR, 3.06; 95% CI, 2.24-4.19), corresponding to the epidemiologic features of VSA. In the registry, carriers without CAD of the risk allele rs112735431 had a strikingly high mortality rate due to acute myocardial infarction during the follow-up period (hazard ratio, 2.71; 95% CI, 1.57-4.65; *P* = 3.3 × 10^−4^). As previously reported, a possible overlap between VSA and Moyamoya disease was not found.

**Conclusions and Relevance:**

Results of this study suggest that vascular cell dysfunction mediated by variants in the *RNF213* locus may promote coronary vasospasm, and the presence of the risk allele could serve as a predictive factor for the prognosis.

## Key Points

**Question:**

Can the first large-scale genome-wide association studies of vasospastic angina (VSA) identify risk for VSA?

**Findings:**

A genome-wide association study including 5720 cases and 153 864 controls was performed using a large sample size and a nationwide, multicohort study. The *RNF213* variants were associated with an increased risk of vasospastic angina, reaching genome-wide significance across the 3 datasets, with the associations being particularly strong in younger male individuals; carriers of the risk allele rs112735431 without coronary artery disease faced a high mortality rate from acute myocardial infarction during follow-up.

**Meaning:**

Results emphasize the need for early intervention to improve patient outcomes.

## Teaser

This genetic association study evaluated genetic variants associated with increased risk of vasospastic angina.

## Introduction

Vasospastic angina (VSA), triggered by spontaneous coronary artery spasms, can lead to life-threatening complications, particularly in patients with enhanced coronary vasoconstrictive reactivity and reduced vasodilator function.^1,2,3^ Identifying at-risk patients is critical to preventing severe or even lethal outcomes. The prevalence of VSA is unclear, with a study in Japan revealing it affects approximately 40% of patients with angina, potentially higher than estimates in White individuals due to differences in clinical provocation tests.^4,5,6^ The central treatment approach focuses on the use of vasodilators and risk factor management, including smoking cessation, blood pressure control, diabetes management, lipid level reduction, stress level reduction, and alcohol intake abatement.^7^ Additionally, VSA has a suggested correlation with migraines.^8,9^

The physiopathology of VSA has been investigated; however, it remains incompletely understood. Several mechanisms have been reported, including hypercontraction of coronary smooth muscle,^10^ enhanced autonomic nervous system activity,^11,12^ endothelial dysfunction, and increased oxidative stress.^13^ Vasospasm can be triggered by decreased availability of nitric oxide (NO) in the endothelium,^13^ and genetic risk factors for VSA have been reported in genes encoding NO synthase.^14^ Very recently, a Japanese group published a short report^15^ investigating the association of the *RNF213* p.R4810K variant with 66 cases of VSA. A previous study by Martina et al^16^ suggested that decreased RNF213 activity could lead to decreased dimethylarginine dimethylaminohydrolase 1 activity, resulting in the accumulation of dimethylarginine and *N*-methylarginine. This could subsequently decrease NO synthase activity and NO levels,^16^ potentially causing coronary spasm as a pathophysiologic link. However, these genetic studies have been limited to candidate gene analyses and small sample sizes.^14,17,18,19^ A small genome-wide association study^20^ (GWAS) reported no loci exceeding genome-wide significant levels. A previous GWAS^21^ for coronary artery diseases (CADs) or angina pectoris included VSA; however, no stratified analyses have been performed specifically for VSA, to our knowledge. A recent Swedish nationwide study^22^ reported high familial heritability for VSA, highlighting the importance of investigating genetic susceptibility. This study aimed to identify genetic factors associated with VSA, and uncover the specific underlying pathologies of VSA.

## Methods

### Study Participants in the First and Second Datasets

In this study, we selected Japanese patients with VSA from the Biobank Japan Project (BBJ) data repository and controls without CAD. We classified patients recruited between 2002 and 2005 as the first dataset and those recruited between 2006 and 2008 as the second dataset. This approach allowed us to have separate datasets for replicating the findings from the first dataset. Baseline characteristics and time-dependent changes were similar across the first and second set of patients. Samples lacking registration year were added to the replication cohort. The combined set encompassed all patients from both cohorts. The diagnosis of VSA, stable angina pectoris, and myocardial infarction (MI) were made by cardiologists based on the relevant guidelines, including the Japanese Circulation Society (JCS) guidelines for VSA,^7^ JCS Guideline on Diagnosis of Chronic Coronary Heart Diseases,^23^ and JCS Guideline on Diagnosis and Treatment of Acute Coronary Syndrome.^3^ Specifically, VSA is suspected from anginalike attacks at rest, during effort, or both. An ischemic change on electrocardiogram (ECG) confirms VSA. In the absence of ischemic ECG changes, VSA is suspected if (1) attacks occur mainly at night or early morning, (2) there is a significant daily variation in exercise capacity with a decrease in the early morning, (3) attacks can be induced by hyperventilation, and (4) calcium channel blockers (but not β-blockers) can suppress the attacks. Stable angina is suspected when there is confirmation of angina symptoms, exclusion of unstable angina, and evidence of myocardial ischemia during exertion. MI is suspected in the presence of chest symptoms suggesting myocardial ischemia, changes in the ECG, and a transient increase in myocardial biomarkers indicating myocardial necrosis.

### Whole-Genome Genotyping, Quality Control, Whole-Genome Imputation, and GWAS

Patients in the BBJ were genotyped using arrays or a set of arrays. We used our original reference panel and performed imputation (eMethods in Supplement 1). GWAS was performed using a Firth logistic regression model in PLINK, version 2.00a2 AVX2 (open-source software), with the command –glm firth. Sex and the top 10 principal components were included as covariates. We also performed GWAS by adding age, smoking (ever vs never), alcohol consumption, co-occurrence of type 2 diabetes, hyperlipidemia, history of hypertension, low-density lipoprotein (LDL) level, and LDL polygenic risk score as covariates to confirm the association signals while accounting for all confounding factors (eMethods in Supplement 1).

### Additional Datasets

To replicate the GWAS in the first and second datasets, we analyzed VSA cases and control samples from the latest patients in the BBJ recruited between 2013 and 2018. We also performed replication analysis using data from the UK Biobank (UKB Resource 531) (eMethods in Supplement 1).

### Ascertainment Schemes

The first and second datasets were derived from the BBJ first cohort, from 2002 to 2008. The first dataset includes individuals recruited from 2002 to 2005, whereas the second dataset encompasses those from 2006 to 2008. Samples without a recorded registration year were included in the second dataset. The third dataset originated from the BBJ second cohort, collected between 2013 and 2018.

### Case Types

The first and second datasets included patients diagnosed with VSA, stable angina, and MI, in accordance with relevant guidelines. The third dataset focused exclusively on patients diagnosed with VSA, following the same guidelines as the first 2 datasets. It included a subset of patients with information on drug-induced vasospasm, specifically used for sensitivity analysis.

### Effect Size of VSA vs non-VSA CAD

We compared β coefficients and SEs for CAD susceptibility variants between VSA and non-VSA CAD. We conducted a permutation test to ascertain if the β coefficient for patients with non-VSA CAD was greater than that for individuals with VSA (eMethods in Supplement 1). In addition, we conducted a fixed-effect inverse variance–weighted meta-analysis of the *RNF213* locus across the 3 datasets (eMethods in Supplement 1).

### Statistical Analysis

#### Sensitivity Analyses

Because detailed clinical information on VSA was available for some of the patients in the third dataset, we extracted cases positive for drug-induced vasospasm in the third dataset. We analyzed the association between the *RNF213* variant and these cases (eMethods in Supplement 1).

We computed the effect sizes of rs112735431, applying a Firth logistic regression (which incorporated sex and top 10 principal components as covariates) for individuals heterozygous or homozygous for the risk allele, referring to individuals homozygous for the nonrisk allele (1 = noncarriers vs heterozygous for rs112735431 and 2 = noncarriers vs homozygous for rs112735431) (eMethods in Supplement 1). We performed stratified analyses by sex or registered age to assess the association of the lead variant with VSA and explored its interactions with sex or age (eMethods in Supplement 1).

#### Correlation and Survival Analyses

We analyzed the associations of the variants susceptible to Moyamoya disease with VSA to assess the shared direction of associations between VSA and Moyamoya disease. We used BBJ follow-up data, which have been previously reported in detail.^24,25^ In addition, we performed Cox regression analyses (eMethods in Supplement 1).

## Results

A total of 5720 cases (mean [SD] age, 67 [10] years; 2048 female [35.8%]; 3672 male [64.2%]) and 153 864 controls (mean [SD] age, 62 [15] years; 76 502 female [49.7%]; 77 362 male [50.3%]) in 3 datasets were included in this study. We included 5192 cases with VSA and 143 964 controls (first dataset: 3807 cases and 89 690 controls; second dataset: 1385 cases and 54 274 controls) (eTable 1 and eFigure 1 in Supplement 1) in the GWAS. Male sex and history of smoking were observed more frequently in cases, as shown previously.^7,26^

**Figure 1.**
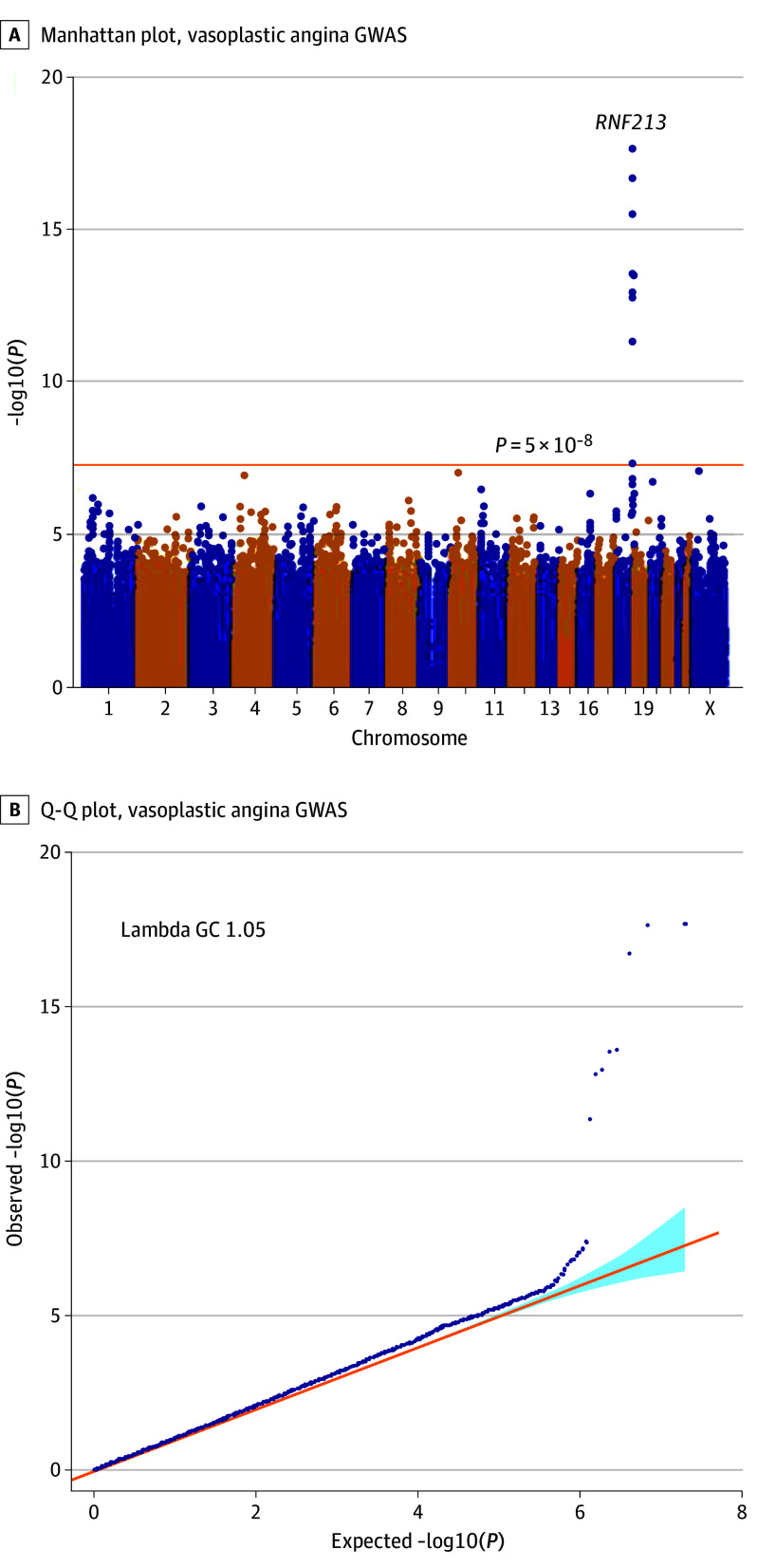
Manhattan and Quintile-Quintile (Q-Q) Plot for Vasospastic Angina Genome-Wide Association Study (GWAS) GWAS results for the combined dataset. Genetic association tests adjusted for sex and principal components 1 to 10. A, Manhattan plot. B, Q-Q plot. The orange line represents the genome-wide significance level (*P* = 5 × 10^−8^). Gene names are shown next to the top loci.

In the first set, GWAS revealed significant loci, including the *RNF213* gene (OR, 2.00; 95% CI, 1.62-2.47; *P* = 1.2 × 10^−10^) (eFigures 1 and 2 and eTable 2 in Supplement 1). In the second set, the *RNF213* gene was the top locus (oR, 2.71; 95% CI, 1.99-3.69; *P* = 2.7 × 10^−10^) (eFigures 1 and 3 and eTable 2 in Supplement 1). In the analysis of the combination of the 2 sets, only *RNF213* showed a significant association with VSA. Additionally, rs112735431, an East Asian–specific^27,28^ rare missense variant in the *RNF213* (p.Arg4810Lys), was the lead variant (OR, 2.18; 95% CI, 1.83-2.59; *P* = 2.0 × 10^−18^) (Figure 1A, Table, and eFigure 4 in Supplement 1). The allele frequencies of rs112735431 in the first and second datasets were 0.016 (cases) and 0.0097 (controls) and 0.020 (cases) and 0.0095 (controls), respectively. Little evidence of substantial inflation in the association statistics was observed (Figure 1B), and we confirmed that strong signals at the locus were robust by altering covariates (Methods in Supplement 1). We also confirmed that no collider bias was introduced. Sensitivity analysis of patients without VSA and with a history of CAD confirmed that the association was not due to control selection. Adding migraine as a covariate did not alter the association, affirming the robustness of our findings. The single-nucleotide variant heritability estimate for the VSA was 3.9% (SE, 1.5%) by using linkage disequilibrium score regression (LDSC). Additionally, the observed heritability was estimated to be approximately 0.8%.

**Table.** Associations Between the Variants at the *RNF213* Locus and Vasospastic Angina in the Present Study

Chr	Variant ID (rs)	First set			Second set			Third set			Meta-analysis		
		AF cases/ controls	OR (95% CI)	*P* value	AF cases/ controls	OR (95% CI)	*P* value	AF cases/ controls	OR (95% CI)	*P* value	AF cases/ controls	OR (95% CI)	*P* value
17	rs112735431	0.016/ 0.010	2.00 (1.62-2.47)	1.2 × 10^−10^	0.020/ 0.010	2.71 (1.99-3.69)	2.7 × 10^−10^	0.026/ 0.008	3.47 (2.29-5.25)	4.4 × 10^−9^	0.018/ 0.010	2.34 (1.99-2.74)	4.4 × 10^−25^
17	rs111321460	0.015/ 0.009	2.09 (1.67-2.60)	6.7 × 10^−11^	0.018/ 0.009	2.79 (2.02-3.85)	5.6 × 10^−10^	0.026/ 0.008	4.01 (2.62-6.15)	1.8 × 10^−10^	0.017/ 0.009	2.47 (2.09-2.92)	5.0 × 10^−26^

Abbreviations: AF, variant allele frequency; Chr, chromosome; ID, identification; OR, odds ratio.

**Figure 2.**
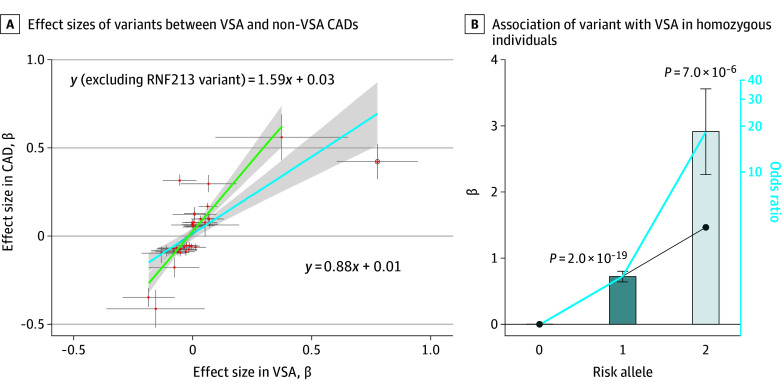
Characteristic Associations of *RNF213* With Vasospastic Angina (VSA) Association analyses results for the combined dataset. A, Comparison of effect sizes of coronary artery disease (CAD)–associated variants between VSA and non-VSA CADs. The bars crossing the red dots represent the 95% CIs of the β coefficients. The dot with a circle is the variants at the *RNF213* locus. The blue line depicts the regression line, with its 95% CI shaded in gray, that includes the *RNF213* variant. The green line represents the regression line, with its 95% CI shaded in gray, without the *RNF213* variant. B, Pronounced association of rs112735431 with VSA in homozygous individuals deviating from the additive model. The effect sizes of rs112735431 on VSA were compared between patients heterozygous and homozygous for the risk allele. Patients homozygous for the nonrisk allele of rs112735431 were used as references. The x-axis shows the number of risk alleles of rs112735431, and the y-axis shows the β coefficients of rs112735431. The bars crossing the black dots in the bar graph represent the SEs of the β coefficients. The diagonal black line represents the assumption of a linear relationship of the effect sizes (based on the β coefficient in heterozygotes). The blue solid line represents the observed increase in the effect sizes in the present study.

Our previous GWAS of CAD (including VSA) revealed an association with the variants at the *RNF213* locus^21^; however, here we noticed that the effect size was different between the 2 phenotypes. Although both CAD and VSA involve underlying pathogenesis in the coronary artery, there may exist distinct aspects that manifest differently among patients. Therefore, we compared the effect sizes of the lead variants in a previous GWAS for CAD (eTable 3 in Supplement 1) between groups with and without VSA and with a history of CAD (eFigure 5 in Supplement 1). Most CAD-associated variants showed the same direction of effects between groups with and without VSA and with a history of CAD (41 of 48 variants, binomial *P* = 6.2 × 10^−7^ and Pearson *r* = 0.72; *P* = 6.6 × 10^−14^) (Figure 2A and eFigure 5 in Supplement 1). However, we observed some exceptions, including obvious non-VSA CAD-specific associations such as rs11066015 in *ACAD10* (OR, 1.37; 95% CI, 1.32-1.41 vs OR, 0.95; 95% CI, 0.88-1.02 and *P* = 4.87 × 10^−79^ vs *P* = .13 for groups without VSA and with VSA, both with a history of CAD, respectively) (eTable 3 in Supplement 1). These findings suggest shared genetic architecture between non-VSA CAD and VSA with some exceptions, supported by a strong genetic correlation (LDSC, *r* = 0.47 and *P* = 9.5 × 10^−5^).

In contrast, the absolute values of the effect sizes of these variants showed clear differences; most of the variants (36 of 41 variants, binomial *P* = 7.8 × 10^−7^) showed higher β coefficients in non-VSA CAD (median, 1.94 folds) than VSA, except for rs112735431 in the *RNF213* gene, which showed a considerably higher β coefficient in VSA than in non-VSA CAD (1.86 folds) (Figure 2A and eFigure 5 in Supplement 1). Other East Asian–specific variants did not follow this trend (eFigure 5 in Supplement 1). These results suggest that rs112735431 could distinctly characterize the manifestations of VSA from those of non-VSA CAD. We also verified the significance of the difference in the strength of association of rs112735431 between VSA and non-VSA CAD by conducting a permutation test. The results showed that the effect sizes for VSA were consistently higher than those for non-VSA CAD (*P* = .001 by generating 1000 random points). Additionally, we consistently observed a distinct effect size of rs112735431 on VSA in the comparison between subgroups of non-VSA CAD (eFigure 6 in Supplement 1) and an increased effect size of rs112735431 in stable angina pectoris compared with MI (eFigure 6 and eTable 3 in Supplement 1).

To further confirm the association between the variants at the *RNF213* locus and VSA, we included 528 VSA cases and 9900 controls and analyzed the associations between VSA and variants at the *RNF213* locus (eTable 1, eFigure 1, and eMethods in Supplement 1). We identified strong associations between the variants at the *RNF213* locus and VSA across the 3 datasets (OR, 4.01; 95% CI, 2.62-6.15; minimum *P* = 1.8 × 10^−10^ in the additional dataset alone and OR, 2.47; 95% CI, 2.09-2.92; minimum *P* = 5.0 × 10^−26^ in the overall study). Additionally, rs112735431 was the second strongest signal in strong LD with the lead intronic variant rs111321460 (*r*^2^ = 0.78) (eFigure 7 in Supplement 1). The subsequent conditional analysis of this locus did not detect any additional independent signals (OR, 0.22; 95% CI, 0.11-0.44; the smallest conditioned *P* = 1.92 × 10^−5^) (eFigure 8 in Supplement 1). We confirmed the VSA-*RNF213* association using UK Biobank data, showing the same signal directions, with case and control frequencies of 0.00017 and 1.40 × 10^−5^, respectively, and a *P* value of .13 due to the small sample size, thereby yielding an OR of 12.0 with an SE of 82.0. Moreover, the haplotype analysis demonstrated that the risk allele of rs111321460 was consistently present in the same haplotype as the risk allele of rs112735431 and that the low-frequency haplotype containing the risk variant of rs112735431 and the reference allele of rs111321460 still showed a trend of association (OR, 1.40; 95% CI, 0.83-2.23; *P* = .17) (eTable 4 in Supplement 1). The deleterious potential of this variant, highlighted by multiple algorithms including a SIFT4G (Sorting Intolerant From Tolerant for Genomes) score of 0.034, a MutationTaster (open-source software) probability score of 1.00, an Mendelian Violation Prediction score of 0.45, an Missense badness Polyphen-2 and Constraint score of 0.16, a Combined Annotation Dependent Depletion phred score of 7.44, and a Deleterious Annotation of Genetic Variants Using Neural Networks score of 0.84, underscores its significant functional implications. Of note, the intronic variant (rs111321460) is associated with significant transcriptional activity, as evidenced by the chromatin state in T cells, including activated CD4-positive T cells. Additionally, single-cell RNA-sequencing datasets from vascular tissues indicate that *RNF213* is expressed in vascular cells, including endothelial cells, smooth muscle cells, macrophages, and T cells. This distribution aligns with the proposed mechanisms underlying VSA, thus supporting our findings.

In addition, we identified a possible pronounced association of the homozygous risk allele of rs112735431 with VSA. When we compared patients carrying homozygous reference alleles to those carrying heterozygous or homozygous rs112735431, the association deviated from linearity (OR, 4.35; 95% CI, 1.18-16.05; *P* = .03) (Figure 2B). Homozygous carriers showed a pronounced association with VSA (OR, 18.34; 95% CI, 5.15-65.22; *P* = 7.0 × 10^−6^) deviating from the additive model, in contrast to heterozygous carriers (OR, 2.05; 95% CI, 1.76-2.40; *P* = 2.0 × 10^−19^). We also confirmed the strong recessive effects by performing the same analyses separately for the combined datasets (first and second datasets) and the additional dataset (third dataset).

**Figure 3.**
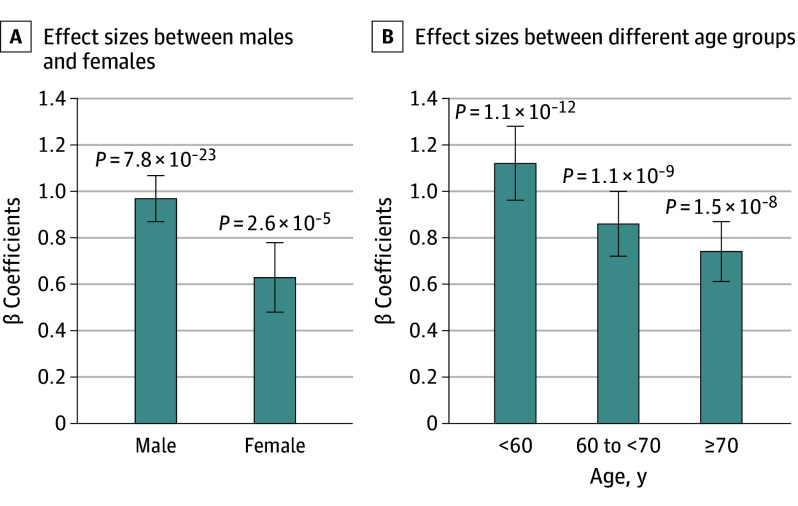
Effect Sizes of rs112735431 on Vasospastic Angina (VSA) Association analyses results using all 3 datasets. A, Effect sizes of rs112735431 are shown between males (3673 cases vs 77 362 controls) and females (2048 cases vs 76 503 controls). Effects sizes are shown among ages younger than 60 years (1073 cases vs 54 389 controls), between 60 and 70 years (1741 cases vs 39 766 controls), and 70 years and older (2379 cases vs 49 810 controls). The bars in the bar graph represent the SEs of the β coefficients.

We also determined the differential association of rs112735431 with VSA according to sex and age. The variant was significantly associated with VSA regardless of sex; however, the effect was much stronger in male than in female participants (male: OR, 2.64; 95% CI, 2.17-3.21 and female: OR, 1.88; 95% CI, 1.40-2.52) (Figure 3A; eTable 5 in Supplement 1). Additionally, there was a significant differential association between rs112735431 and male individuals (χ^2^_1_ = 7.24; *P* = .007). The variant rs112735431 demonstrated a tendency for stronger effect sizes in young age groups (OR, 3.06; 95% CI, 2.24-4.19) (Figure 3B and eTable 5 in Supplement 1). However, further investigations are needed to unravel the mechanisms underlying these observed sex differences. Additionally, rs112735431 showed an association (with a strong effect size) with cases positive for drug-induced vasospasm (eMethods and eTable 6 in Supplement 1). These associations were robust regardless of the covariates.

As rs112735431, an Asian-specific rare deleterious variant (p.Arg4810Lys), is a well-known causal variant of Moyamoya disease (a disease of occlusion of the cerebral vasculature causing intracranial hemorrhage with unknown etiology),^29,30^ we analyzed the potential co-occurrence and confounding of Moyamoya disease in this study. We analyzed 10 susceptibility variants of Moyamoya disease from the Chinese population to assess shared associations with VSA.^31^ We identified 31 patients with Moyamoya disease of the total number of patients in the GWAS, which is a reasonable number considering its prevalence^31,32,33,34^ (eAppendix in Supplement 1); however, we did not observe statistically significant enrichment of Moyamoya disease in patients with VSA (only 2 patients had VSA; OR, 1.91; 95% CI, 0.22-7.56; Fisher *P* = .28). We also failed to identify a common direction of association between VSA and Moyamoya disease in the 10 susceptibility variants (excluding the variants at the *RNF213* locus) and Moyamoya disease (5 of 10 variants showing the same risk allele) (eTable 7 and eAppendix in Supplement 1).^31^ Therefore, it is unlikely that Moyamoya disease confounded the findings.

**Figure 4.**
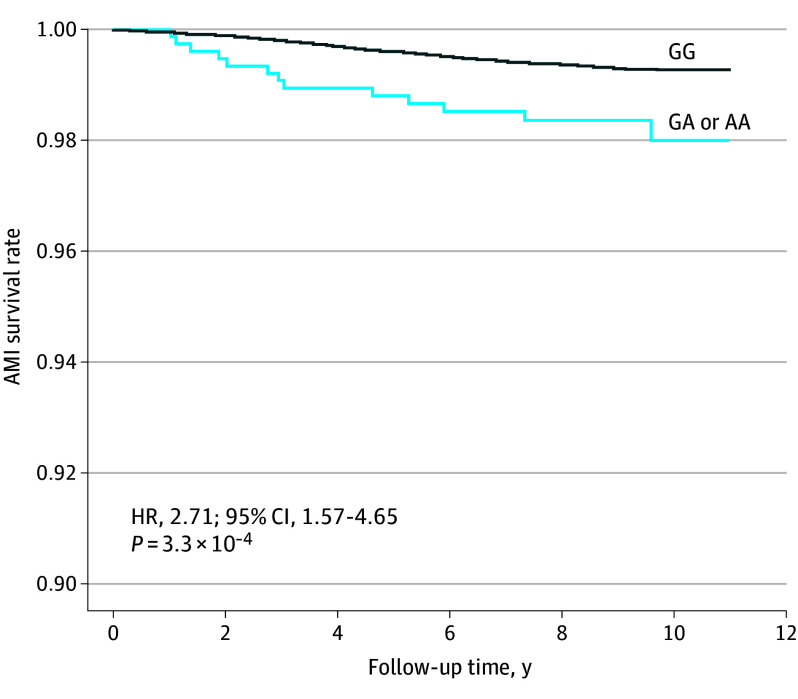
High Mortality Rate of Acute Myocardial Infarction (AMI) in Individuals Carrying rs112735431 Kaplan-Meier survival curve for patients with and without risk alleles of rs112735431 who die due to AMI registered at the Biobank Japan Project, excluding those registered for cancers and cardiac diseases. The x-axis represents the follow-up years of the patients, and the y-axis represents survival rates for AMI. The solid curve represents outcomes in patients without the risk allele (A) of the variant in the VSA. The blue curve represents those without the risk alleles. AA indicates homozygous individuals; GA, heterozygous individuals; GG, wild-type individuals; HR, hazard ratio; VSA, vasospastic angina.

We further investigated the association between rs112735431 and mortality rates due to acute MI (AMI) in the follow-up data from the BBJ. To avoid potential confounding effects of CAD and malignancies as baseline diseases, we excluded all patients with CAD (either VSA or non-VSA CAD) or malignancies from the registry. Comparison of the fatal AMI survival rates between carriers of the risk allele rs112735431 and noncarriers, based on a 10-year-follow-up, revealed a high mortality rate in patients with AMI carrying the risk allele (hazard ratio [HR], 2.71; 95% CI, 1.57-4.65; *P* = 3.3 × 10^−4^) (Figure 4). Additionally, when considering sudden death as the outcome instead of fatal AMI, the results showed an HR of 1.96 (95% CI, 0.28-14.0). Despite the pronounced HR, the statistical power was insufficient to achieve a significant *P* value (*P* value of .50), especially given that only 36 patients experienced sudden death. However, this underscores the potential risk association for carriers susceptible to sudden death. These results suggest that the presence of the risk of allele rs112735431 could potentially be a predictive factor for the prognosis. Of note, we have added eTable 8 in Supplement 1 to clearly indicate which datasets were used in each analysis.

## Discussion

This study presents the first, to our knowledge, large-scale GWAS of VSA, identifying the *RNF213* locus, including a population-specific missense variant known for vascular dysfunction, as a possible risk factor associated with VSA. The locus showed a strong association with the development of VSA and the mortality rate due to AMI, providing new insights into the underlying pathophysiology and mechanisms of VSA.

In the present study, rs112735431 showed an association with sex in VSA; rs112735431 exhibited a strong association with male patients, which might explain the differences in the prevalence of VSA between males and females. We also observed a trend of strong effect sizes in young groups. As VSA develops in relatively young people compared with non-VSA CAD, our findings of enhanced associations in young groups may highlight the differences in the underlying mechanisms between VSA and non-VSA CAD.^35^ Additionally, we observed increased mortality associated with rs112735431 in patients without CAD, suggesting that rs112735431 may tag a genetic region, which is associated with increased mortality in patients without CAD. We also observed that the effect sizes of rs112735431 on susceptibility to VSA and future death due to AMI were comparable. The strong association between rs112735431 and young age and sex (male) suggests that the presence of rs112735431 may be associated with a higher risk of fatal AMI in young male individuals.

The haplotype analyses suggest an effect of rs112735431 alone and a possible combinatory effect of both variants, supported by the fact of its missense function and important roles in the maintenance of vascular cells.^34^ Additionally, homozygous carriers showed a pronounced association with VSA, which suggests that the dysfunction of *RNF213* due to rs112735431 in homozygous carriers may have a substantial association with the development of VSA. A missense variant of the *RNF213* gene, rs112735431, has been reported to be associated with CADs, including angina pectoris.^21,36^ Recently, functional studies for this variant have begun to emerge. Induced pluripotent stem cell–derived vascular endothelial cells from patients with Moyamoya disease with the *RNF213* variant show decreased angiogenic activities,^37,38^ which could be attributed to endothelial dysfunction in cardiovascular systems.^13^ This observation aligns with the potential contribution of the mechanisms of VSA, with vascular smooth muscle hyperreactivity seen as the primary mechanism.^39,40^ Another study^41^ has shown that *RNF213* attenuates WNT/calcineurin/NFAT signaling. The WNT/calcineurin pathway plays important roles during heart development, and WNT9b plays an important role in coronary artery formation via the β-catenin pathway.^42^ Therefore, these findings are consistent with ours. Moreover, as stated previously, the findings from a previous study by Martina et al^16^ could possibly lead to the hypothesis of a potential pathophysiologic link between *RNF213* variants and vasospasm. Although this suggested hypothesis is intriguing, the findings of the study require validation in vitro. Previous studies have identified genetic variants linked to VSA.^17^ Our study replicated some of these, notably finding consistent associations with rs10498345, rs5963409 in the *OTC* gene, and rs9349379 in the *PHACTR1* gene, the latter known to be associated with elevated levels of endothelin 1.^17,18,20^ However, our findings diverged for ALDH2*2 and the *eNOS* T-786C variant, possibly due to the use of non-GWAS methodologies and smaller sample sizes in prior research.^14,19^ Despite these discrepancies, the successful replication of other variants supports our study’s credibility and underscores that the interplay of these factors likely is associated with the development of VSA.

We found that Moyamoya disease unlikely confounds VSA cases, backed by reviews doubting coincidental CAD risks, although not assessing VSA directly. VSA was more common in males than in females, with a female to male ratio of 1.8:1 in Moyamoya disease. Only *RNF213* variants showed significant associations, marking distinct pathologies from VSA. The increasing evidence related to the *RNF213* variant suggests that the missense variant (or its haplotype) has a wide-ranging association with vascular dysfunction.^43,44^ Future studies should further explore the risk of this variant for VSA and other vasculopathies.

### Limitations

Our research has limitations, notably the absence of detailed diagnostic criteria met at the BBJ, a challenge shared with many biobanks. Previous studies have taken similar approaches to ours, including the use of *International Statistical Classification of Diseases and Related Health Problems* codes.^45^ Despite the challenges encountered, our study successfully identified and replicated significant genetic loci associated with VSA. The consistency of our findings across 3 distinct datasets, coupled with sensitivity analyses focused on samples with positive provocation test results and corroborated by UK Biobank data (as well as the very recently published study by a Japanese group^15^), strongly supports the veracity of our identified signals. In the future, more detailed analyses should incorporate symptoms, subtypes of spasms, angiography data, details of coronary atherosclerosis, details of fatal AMI and sudden death, and results of negative spasm provocation tests. Additionally, exploring narcotic use may offer insights, as drug-induced vasospasm cases could be underreported. Our findings concerning the higher incidence of vasculopathies, such as VSA, in patients with Moyamoya disease were not statistically significant. Therefore, the results are inconclusive due to the limited sample sizes. The associations of Moyamoya disease and sudden death necessitate more extensive investigations. We also need to mention that this study lacked mechanistic details on how the risk variant or *RNF213* influences VSA, calling for more research in cellular and/or animal models.

## Conclusions

Results of this study suggest that the *RNF213* variants were associated with an increased risk of VSA, reaching genome-wide significance across the 3 datasets, with the associations being particularly strong in younger male individuals. Carriers of the risk allele rs112735431 without coronary artery disease faced a high mortality rate from AMI during follow-up. Results emphasize the need for early intervention to improve patient outcomes. Our results suggest that *RNF213* was associated with the pathophysiology of VSA, and this study may provide insights for developing personalized interventions to prevent lethal outcomes.

## Notes

Supplement 1.
eMethods.
**eAppendix.** Co-Occurrence of Moyamoya Disease and VSA and the Possibility of Confounding Moyamoya Disease in the Present Results **eFigure 1.** Summary of Patients in the Present Study **eFigure 2.** Genome-Wide Association Study for VSA in the First Dataset **eFigure 3.** Genome-Wide Association Study for VSA in the Second Dataset **eFigure 4.** Associations in the *RNF213* and VSA in the Combined Datasets **eFigure 5.** Effect Sizes of the CAD-Associated Variants Between VSA and CADs **eFigure 6.** Effect Sizes of rs112735431 Between VSA and the CAD Subtypes **eFigure 7.** Locus zoom Plot of the Results of the Meta-Analysis **eFigure 8.** Locus Zoom Plot of the Conditional Analyses of rs112735431 **eTable 1.** Baseline Characteristics of the Patients Included in This Study **eTable 2.** Associations With VSA Identified in the First or Second Datasets **eTable 3.** Comparison of Effect Sizes of CAD-Associated Variants Among Subgroups of CADs **eTable 4.** Haplotype Analysis of the Variants at the *RNF213* Locus **eTable 5.** Stratified Analysis of the Association Between *RNF213 *and Vasospastic Angina Based on Sex and Age **eTable 6.** Association Between the *RNF213* Locus and Patients With Vasospastic Angina Positive for Drug-Induced Vasospasm **eTable 7.** Nonshared Effect Direction of Moyamoya Disease-Associated Variants Between Vasospastic Angina and Moyamoya Disease **eTable 8.** Overview of the Included Datasets in Each Analysis
eReferences

Supplement 2. **Nonauthor Collaborators.** The Biobank Japan Project.

Supplement 3.
Data Sharing Statement.
