# Supplementary material for: RNF213 Variants, Vasospastic Angina, and Risk of Fatal Myocardial Infarction
Source: JAMA Cardiol. 2024 Jun 18;9(8):723–31. doi: 10.1001/jamacardio.2024.1483 (PMC11195602; doi:10.1001/jamacardio.2024.1483)
Supplement: Supplement 1. — eMethods. eAppendix. Co-Occurrence of Moyamoya Disease and VSA and the Possibility of Confounding Moyamoya Disease in the Present Results eFigure 1. Summary of Patients in the Present Study eFigure 2. Genome-Wide Association Study for VSA in the First Dataset eFigure 3. Genome-Wide Association Study for VSA in the Second Dataset eFigure 4. Associations in the RNF213 and VSA in the Combined Datasets eFigure 5. Effect Sizes of the CAD-Associated Variants Between VSA and CADs eFigure 6. Effect Sizes of rs112735431 Between VSA and the CAD Subtypes eFigure 7. Locus zoom Plot of the Results of the Meta-Analysis eFigure 8. Locus Zoom Plot of the Conditional Analyses of rs112735431 eTable 1. Baseline Characteristics of the Patients Included in This Study eTable 2. Associations With VSA Identified in the First or Second Datasets eTable 3. Comparison of Effect Sizes of CAD-Associated Variants Among Subgroups of CADs eTable 4. Haplotype Analysis of the Variants at the RNF213 Locus eTable 5. Stratified Analysis of the Association Between RNF213 and Vasospastic Angina Based on Sex and Age eTable 6. Association Between the RNF213 Locus and Patients With Vasospastic Angina Positive for Drug-Induced Vasospasm eTable 7. Nonshared Effect Direction of Moyamoya Disease-Associated Variants Between Vasospastic Angina and Moyamoya Disease eTable 8. Overview of the Included Datasets in Each Analysis eReferences [file jamacardiol-e241483-s001.pdf]

Supplementary Online Content

Hikino K, Koyama S, Ito K, et al. *RNF213* variants, vasospastic angina, and risk of fatal myocardial infarction. *JAMA Cardiol*. Published online June 18, 2024. doi:10.1001/jamacardio.2024.1483

eMethods.

eAppendix. Co-Occurrence of Moyamoya Disease and VSA and the Possibility of Confounding Moyamoya Disease in the Present Results

eFigure 1. Summary of Patients in the Present Study

eFigure 2. Genome-Wide Association Study for VSA in the First Dataset

eFigure 3. Genome-Wide Association Study for VSA in the Second Dataset

eFigure 4. Associations in the *RNF213* and VSA in the Combined Datasets

eFigure 5. Effect Sizes of the CAD-Associated Variants Between VSA and CADs

eFigure 6. Effect Sizes of rs112735431 Between VSA and the CAD Subtypes

eFigure 7. Locus zoom Plot of the Results of the Meta-Analysis

eFigure 8. Locus Zoom Plot of the Conditional Analyses of rs112735431

eTable 1. Baseline Characteristics of the Patients Included in This Study

eTable 2. Associations With VSA Identified in the First or Second Datasets

eTable 3. Comparison of Effect Sizes of CAD-Associated Variants Among Subgroups of CADs

eTable 4. Haplotype Analysis of the Variants at the *RNF213* Locus

eTable 5. Stratified Analysis of the Association Between *RNF213* and Vasospastic Angina Based on Sex and Age

© 2024 Hikino K et al. *JAMA Cardiology*.

eTable 6. Association Between the *RNF213* Locus and Patients With Vasospastic Angina Positive for Drug-Induced Vasospasm

eTable 7. Nonshared Effect Direction of Moyamoya Disease-Associated Variants Between Vasospastic Angina and Moyamoya Disease

eTable 8. Overview of the Included Datasets in Each Analysis

eReferences

© 2024 Hikino K et al. *JAMA Cardiology*.

eMethods.

Study participants

The BioBank Japan Project (BBJ) is a national biobank project that enrolled patients from 2002 to 2008 and a nationwide hospital-based genome cohort.[1] The BBJ consists of DNA samples and clinical data related to 47 target diseases from approximately 200,000 patients.[1]

In this study, we selected patients with VSA from the BBJ data and controls without CADs. The diagnosis of VSA, stable angina pectoris, and myocardial infarction (MI) were made by the cardiologists based on the relevant guidelines.

The medical coordinators in BBJ collected clinical information from the medical records of patients which were documented by physicians-in-charge (BioBank Japan, https://biobankjp.org/english/pdf/english.pdf). We also selected patients with stable angina pectoris, MI, and CADs (stable angina pectoris or MI), which were included among the 47 target diseases in the BBJ but excluded those with VSA from the cases to avoid overlapping in comparative analysis with VSA (eFigure 1 and eTable 1).

The study's protocol was approved by the ethical committees (Approval No. 17-17-16[8]) at the Institute of Medical Sciences, The University of Tokyo (Tokyo, Japan), and the RIKEN Center for Integrative Medical Sciences (Yokohama, Japan). All patients recruited provided written informed consent. All relevant ethical regulations were complied with throughout this study.

Whole-genome genotyping and quality control (QC)

BBJ patients were genotyped using arrays or a set of arrays, namely: (1) a combination of Illumina Infinium Omni Express and Human Exome, (2) Infinium Omni Express Exome v1.0, and (3) Infinium Omni Express Exome v1.2.

QC was performed for samples and variants as described in our previous GWAS using data from the BBJ.[2,3] Refer to the eMethods for details of QC. Briefly, we used Plink v.1.9 software.[4] The exclusion criteria for QC for samples were set as follows: (1) call rates of < 0.98, (2) identical to others genetically, (3) genotypic and phenotypic sex mismatch, and (4) outliers from the East Asian cluster which were identified using a principal component analysis, for which we used the three major reference populations (Africans, Europeans, and East Asians) in the International HapMap Project and samples that were genotyped.[5,6] The exclusion criteria for QC for variants were set as follows: (1) call rate of < 0.99, (2) P-values for Hardy–Weinberg

© 2024 Hikino K et al. *JAMA Cardiology*.

equilibrium (which posits that the proportion of alleles and genotypes remains constant from one generation to the next) of $< 1.0\times10^{-6}$, and (3) number of heterozygotes < 5.

Whole-genome imputation

We used our original reference panel that was recently developed using whole-genome sequencing (WGS) data of 3,256 Japanese individuals in the BBJ and 2,504 individuals in the 1000 Genomes Project (1KG; phase3v5) to achieve higher imputation accuracy for the Japanese population. Successful identification of population-specific variants using this reference panel has been reported;[3] please refer to Terao, et al. for details on the development of the reference panel ('Reference panel using whole genome sequencing data in Japanese Population', manuscript in submission). A total of 5,760 individuals were included in the reference panel, which comprised 72,406,123 autosomal variants and 3,252,444 chromosome X variants. Upon imputation, we excluded samples which overlapped with those in the reference panel, as previously described.[2,3] We mapped all variants to the National Center for Biotechnology Information (NCBI) build 37 (hg19). Notably, the R-squared value, a statistical metric indicating the quality and reliability of imputation, for rs112735431 was 0.71.

GWAS

GWAS was performed using a Firth logistic regression model in PLINK (version 2.0) (Table 1) in order to stabilize the estimation of effect sizes for rare variants, with inclusion of sex and the top ten principal components (PC) as covariates. We also performed GWAS by adding all the confounding factors as described in the Methods in the main text, to confirm the association signals while accounting for all confounding factors. We used KING software (version 2.2.5) to estimate the kinship coefficients for pairwise relationships and then excluded a 2nd-degree kinship.[7] The following variants were excluded for GWAS: minor allele frequencies (MAF) < 0.005 and imputed with R-squared value < 0.3. Manhattan plots were drawn using the R software (version 4.0.2). Statistical significance for associations was set P at $< 5.0 \times 10^{-8}$. We defined a locus as a significantly associated locus when the genomic region was within ±1 megabase (Mb) from lead variants. Regional association plots were generated using LocusZoom (version 1.2).[8] We considered there to be little evidence of substantial inflation when the estimated inflation factor λ_{GC} was <1.05.

For conditional analysis, we employed genome-wide complex trait analysis–conditional and joint analysis (GCTA-COJO) until all significant associations were accounted for.[9]

For calculating the polygenic risk scores (PRS) for LDL, we utilized the GWAS results from our paper (Koyama, Liu, and Koike et al, Accepted by Nature Genetics 2023). In brief, we conducted a GWAS using BOLT-LMM software package after normalizing the LDL levels. We then constructed the PRS for LDL using a pruning and thresholding method.[10] Using the GWAS result, the PRS was generated by summing risk alleles, which were weighted by the natural logarithms of the odds ratios from the GWAS by PLINK (version 1.9). We employed an additional dataset (the third dataset) as the discovery dataset to extract the odds ratio and then applied it to calculate the PRS using the combined 1st and 2nd datasets.

Importantly, since our study is a GWAS focusing on VSA, we included all individuals diagnosed with VSA in the case group, regardless of any MI comorbidities. Additionally, we verified the consistent association signals from the GWAS by excluding the 449 VSA cases that also had MI.

Heritability estimation

Liability-scale heritability was estimated in our GWAS results with linkage disequilibrium score regression (LDSC, version 1·0·0) to offer a more comprehensive view of genetic contribution across the entire population, not just those with the disease,[11] excluding variants in the human leukocyte antigen (HLA) region (chromosome 6: 26–34Mb) and calculating heritability z scores as well in order to assess the reliability of heritability estimation. We assume the prevalence of VSA around 2.1% based on prevalence of CAD and reported fraction of VSA in CAD.[12-14] Subsequently, observed heritability was also estimated.

Additional data setss

To replicate the GWAS in the first and second data sets, we analysed 528 VSA cases and 9,900 control samples from the latest patients in BBJ recruited between 2013-2018. We confirmed that there was no sample overlap between the datasets. The diagnosis of the subjects was identical to that in the first and second data sets. There were no differences in VSA ascertainment compared with the first and second datasets. We extracted results in the variants at the *RNF213* locus from the association results in which genotyping was performed using the Infinium Asian Screening Array (ASA) v1.0 BeadChip and quality control, imputation, and association studies, which were conducted as described above. Additionally, we utilized data from the UK Biobank (UKB Resource 531) to perform replication analysis. Our selection process involved 327 cases identified with ICD-10 code I20.1 and 2,529 controls, which were randomly

© 2024 Hikino K et al. *JAMA Cardiology*.

selected, after excluding individuals with coronary artery diseases identified by ICD-10 codes I20 through I25, using the same methods as with the third dataset.

Associations between the *RNF213* and patients with VSA positive for drug-induced vasospasm

Because detailed clinical information on VSA was available for some of the patients in the third data set, we focused on drug-induced vasospasm (induced by either acetylcholine or ergonovine) and extracted a total of 244 cases positive for drug-induced vasospasm in the third data set. We analysed the association between the *RNF213* variant and these cases using the same control (9,900) group in the third data set by applying a Firth logistic regression model.

Candidates of a causal variant and functional annotation of the variants

We focused on variants in strong LD with lead variant (r^2>0.7) and used ANNOVAR[15] to annotate the variants.

Comparison of effect sizes between VSA and non-VSA CADs

We compared the beta coefficients and standard errors (SE) of the variants associated with susceptibility to CADs (defined by the variants in Koyama et al),[12] between VSA and non-VSA CADs (eFigure 1). We calculated the correlation of effect sizes between VSA and non-VSA CADs to demonstrate the shared genetic architectures between the two disease categories. In addition, we analysed the consistent direction of the associations between VSA and non-VSA CADs using a binomial test to determine if observed proportions are consistent with expected proportions. We also compared the absolute value of effect sizes using a two-sided binomial test to analyse whether the absolute values of effect sizes are greater in non-VSA CADs than in VSA or vice versa.

Permutation test

We produced 1000 random data points using a normal distribution, centered around the mean and standard deviation of the beta coefficient. Then, we compared these values between patients with VSA and non-VSA CADs. We tested the alternative hypothesis that the beta coefficient for patients with non-VSA CAD is greater than that for those with VSA by tallying the test results.

Evaluation of genetic correlations

© 2024 Hikino K et al. *JAMA Cardiology*.

We estimated genetic correlations, using LDSC, between the results of the GWAS for patients with VSA and non-VSA CADs, using East Asian LD scores from 1 KG, where we excluded variants in the HLA region (chromosome 6:26–34Mb).[11,16] We randomly divided the controls into two groups (as controls for VSA and non-VSA CADs) to exclude any overlapping samples in the two datasets.

Meta-analysis and conditional analyses for the variants at the *RNF213* locus

We subsequently conducted a fixed-effect inverse variance-weighted meta-analysis of the *RNF213* locus across the three datasets using METAL software (Released on May 5, 2020).[17] This method gives more importance to studies with higher precision by using the inverse of their variance as weights. In terms of conditional analysis, GCTA-COJO was used until no significant associations were identified. All downstream analyses of *RNF213* were conducted based on the meta-analysis results unless stated otherwise.

LD structure and haplotype analysis

We used Haploview software (version 4.2) to estimate the LD between variants, construct haplotypes, and conduct haplotype association analysis in the variants at the *RNF213* locus.

Evaluation of effects size of rs112735431 in heterozygote or homozygote individuals

We computed the effect sizes of rs112735431, using applying a Firth logistic regression which incorporated sex and top ten PC as covariates, for individuals heterozygous or homozygous for the risk allele, referring to individuals homozygous for the non-risk allele (1.non-carriers vs heterozygote and 2.non-carriers vs homozygote subjects of rs112735431). Among the VSA cases, 5,541 were non-carriers, 175 were heterozygous carriers, and 4 were homozygous carriers. In the control group, 143,933 were non-carriers, 2,247 were heterozygous carriers, and 6 were homozygous carriers. To estimate the departure of effect sizes in homozygotes of rs112735431 from the additive model, we used a binary variable of homozygote status in logistic regression in addition to covariates and rs112735431 genotypes, as previously described.[18]

Stratified analyses and validation of the associations between *RNF213* and VSA using additional covariates

© 2024 Hikino K et al. *JAMA Cardiology*.

We performed stratified analyses to determine the impact of the lead variant on VSA based on sex or registered age. For the sex-stratified analysis, we divided the control groups into men and women and included them in the association analysis. Age was categorised into three groups: (1) < 60 years, (2) 60–70 years, (3) ≥70 years, and included age-matched control groups. We applied a Firth logistic regression to examine the association between the lead variant and VSA. Additionally, we investigated the interactions between the lead variant for VSA and sex or age by logistic regression analyses, adding the top 10 PC as covariates, and using R (version 4.0.2). We also performed analyses including GWAS by adding smoking, alcohol consumption, co-occurrence of type 2 diabetes, hyperlipidaemia, and histories of hypertension as covariates in addition to the ones listed above. To evaluate the associations between variants and sex or age, we simultaneously adjusted for the variant and sex or age. If any association existed, we confirmed it by comparing two logistic regression models by the Chi-square test using analysis of variance (ANOVA). The first model was the one we used to observe the interaction, as described above. For the second one, the explanatory variable was the risk allele counts of the lead variant for VSA and sex or age, and the response variable was the affected status of VSA.

DNase I-hypersensitive sites data

We obtained the definitions of DNase I-hypersensitive sites (DHSs) from ENCODE3 projects using bulk samples [19] and saw the overlapping locations. RegulomeDB was also utilized for annotation.[20]

Cell-type specific expression of *RNF213* by querying single-cell RNA-seq

We utilized the CELLxGENE Explorer[21] to investigate the cell-type specific expression of *RNF213*.

In Silico evaluation of the missense variant rs112735431 using multiple algorithms

We conducted an in-depth in silico analysis of the missense variant rs112735431, utilizing a comprehensive suite of amino acid prediction algorithms by dbNSFP.[22,23] These included SIFT, SIFT4G, Polyphen2 HDIV, Polyphen2 HVAR, LRT, MutationTaster, MutationAssessor, FATHMM, PROVEAN, VEST4, MetaSVM, MetaLR, MetaRNN, M-CAP, REVEL, MutPred, MVP, MPC, PrimateAI, DEOGEN2, BayesDel, ClinPred, LIST-S2, Aloft, CADD, DANN, fathmm-MKL, fathmm-XF, EIGEN, EIGEN-PC, GenoCanyon, integrated fitCons, LINSIGHT, GERP++, phyloP100way, phyloP30way, phyloP17way, SiPhy, and bStatistic. This array of

© 2024 Hikino K et al. *JAMA Cardiology*.

predictive tools was selected to comprehensively assess the variant's potential impact on protein function and its association with disease phenotypes.

Correlation analysis between Moyamoya disease susceptibility variants and VSA

We obtained 10 Moyamoya disease-susceptibility variants reported in the Chinese population[24] and analysed the associations of these variants with VSA to assess the shared direction of associations between VSA and Moyamoya disease. We extracted association results from the combined GWAS data (first and second data sets) and defined P-values of 0.05/10 (after Bonferroni's correction) as significant.

Survival analysis for patients in the BBJ based on the presence of the risk allele of the lead variant in the *RNF213* region for VSA

We used BBJ follow-up data, which have been previously reported in detail. [1] In brief, the follow-up data was survival data collected for approximately 140,000 participants. Medical coordinators identified BBJ participants who had not visited the hospital for more than a year based on the 2010 medical record survey. Medical coordinators recorded whether the participants had died based on a copy of the resident card obtained from the local government. Vital statistics were obtained from the Statistics and Information Department of the Ministry of Health, Labour, and Welfare in Japan, and the cause of death was identified according to the ICD-10 code by matching personal information. Therefore, data were obtained from the national registry, and the diagnosis was based on the ICD-10 codes given by physicians. These data were also used in previous studies, including those on CADs,[12,25] where reasonable associations between CAD risk and future death from CAD were reported.

We analyzed the mortality of patients with ICD-10 codes I21 (acute myocardial infarction, AMI) and R96 and I46.1 (sudden death) using the Cox proportional hazard model. A total of 61,387 patients from the BBJ registry, who were free from cardiac diseases and cancers, were included in this study. Our objective was to compare the mortality between patients with and without rs112735431. We performed Cox regression analyses. Age, sex, smoking status, baseline disease status (target diseases in the BBJ including type 2 diabetes and hyperlipidaemia), and genotyping arrays were used as covariates. As an additional analysis, we also applied the model by adding other risk factors for CADs (alcohol consumption and history of hypertension) to covariates (at the expense of decreased sample size).

Data availability

© 2024 Hikino K et al. *JAMA Cardiology*.

The full GWAS results can be accessed through the website of the Japanese ENcyclopedia of GEnetic Associations by Riken (JENGER, http://jenger.riken.jp/en/).

© 2024 Hikino K et al. *JAMA Cardiology*.

eAppendix. Co-occurrence of Moyamoya disease and VSA and the possibility of confounding Moyamoya disease in the present results.

We analysed whether patients with VSA in the present study also suffer from Moyamoya disease and whether the association of *RNF213* is confounded by the co-occurrence of Moyamoya disease.

Moyamoya disease was not included in the 47 target diseases for which the BBJ participants were recruited. In the text search of clinical information available on the patients of the BBJ, we found 31 patients with Moyamoya disease in the dataset, of which only 2 had VSA. This is a reasonable number considering the low prevalence of Moyamoya disease[26] and elderly participants recruited by the BBJ while visiting hospitals.[1] We did not observe statistically significant enrichment of Moyamoya disease in the VSA group (Fisher's exact test, P=0.29, odds ratio=1.91, 95% confidence interval 0.22-7.56).

Next, we provided genetic evidence to support the difference between the two diseases. A recent study has reported a total of 10 risk loci for Moyamoya disease (other than *RNF213*) by GWAS.[24] However, none of the 10 lead variants showed nominally significant associations with VSA in the current study (P>0.05/10) (eTable 7). Importantly, we did not even observe a trend of the consistent direction of associations in the 10 loci between the two diseases (5/10, 50%) showed the same direction (which is the same probability as a random coin toss). These results suggest that the patients with VSA in the current study did not suffer from Moyamoya disease, and the association results were not confounded by possible Moyamoya disease.

These results also suggest that VSA is distinct from Moyamoya disease. Epidemiologically, VSA and Moyamoya disease do not co-occur frequently. A recent review paper has summarized the coincidental risks of Moyamoya disease and CAD and concluded that there is no epidemiological evidence of co-occurrence and coincidence would be unlikely.[27] This might be supported by epidemiological data for each disease; VSA is more prevalent in men than in women[28] (also supported by our data sets), whereas Moyamoya disease is a women-prevalent disease (with a women-to-men ratio of 1:1.8).[26]

In addition, considering the allele frequency of rs112735431 of 0.2% in the East Asian population, approximately 520,000 individuals are carrying the variant in Japan [130

million Japanese population × ($0.002^2 + 2 \times 0.002 \times 0.998$) = 0.52 million]. We can estimate the total number of patients with Moyamoya disease as 4,000 in Japan (based on the prevalence of Moyamoya disease and population in Japan), indicating that only about 0.6% of the individuals carrying the variants suffer from Moyamoya disease (assuming up to 80% of patients with Moyamoya disease carrying this variant,[29] 4000 * 0.8 =3200 patients with Moyamoya disease carrying this variant, and there are 0.52 million carriers). Therefore, it is not surprising that *RNF213* p.Arg4810Lys is associated with disease(s) other than Moyamoya disease, and the two diseases do not frequently co-occur.

© 2024 Hikino K et al. *JAMA Cardiology*.

eFigure 1. Summary of patients in the present study.

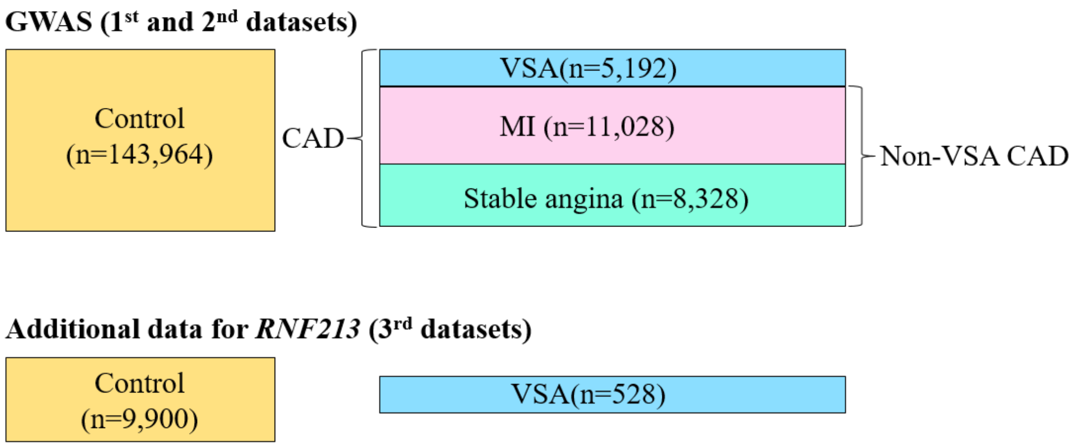

This figure helps to intuitively understand the patients in this study, together with eTable 1. There was no sample overlap among the subtypes of CADs, namely, VSA, MI, and stable angina pectoris. We defined MI and stable angina pectoris as non-VSA CAD cases. The same control patients were used for VSA (1st and 2nd data sets), MI, and stable angina pectoris. Additional data from 528 VSA and 9,900 controls were recruited to confirm the findings of *RNF213*. CAD, coronary artery disease; VSA, Vasospastic angina; MI myocardial infarction.

© 2024 Hikino K et al. *JAMA Cardiology*.

eFigure 2. Genome-wide association study for VSA in the first data set.

Genetic association tests adjusted for sex and principal components 1 to 10.

(a)

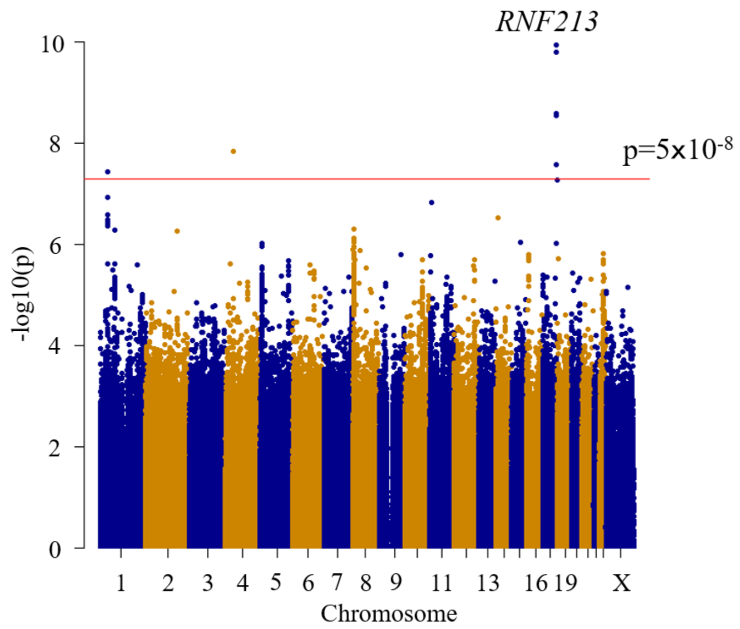

(b)

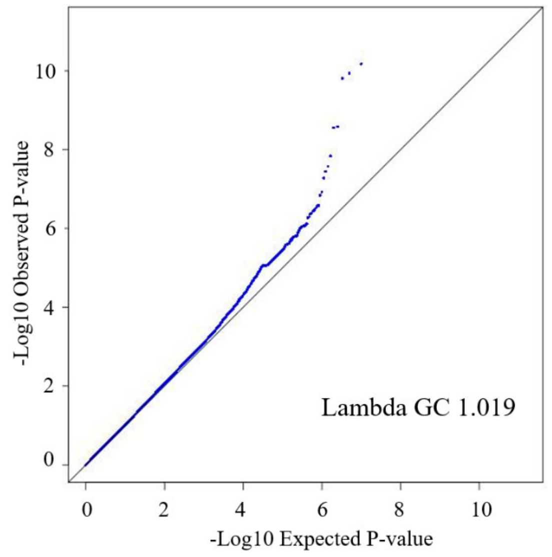

© 2024 Hikino K et al. *JAMA Cardiology*.

(a) Manhattan plot. Results are plotted as –log10 p-values on the y-axis by position on the chromosome (x-axis, NCBI build 37). The red line represents the genome-wide significance level ($P=5\times 10^{-8}$). Gene names are shown next to the top loci.

(b) Q–Q p plot showing observed versus expected P-values. GC, genomic control.

© 2024 Hikino K et al. *JAMA Cardiology*.

eFigure 3. Genome-wide association study for VSA in the second data set.

Genetic association tests adjusted for sex and principal components 1 to 10.

(a)

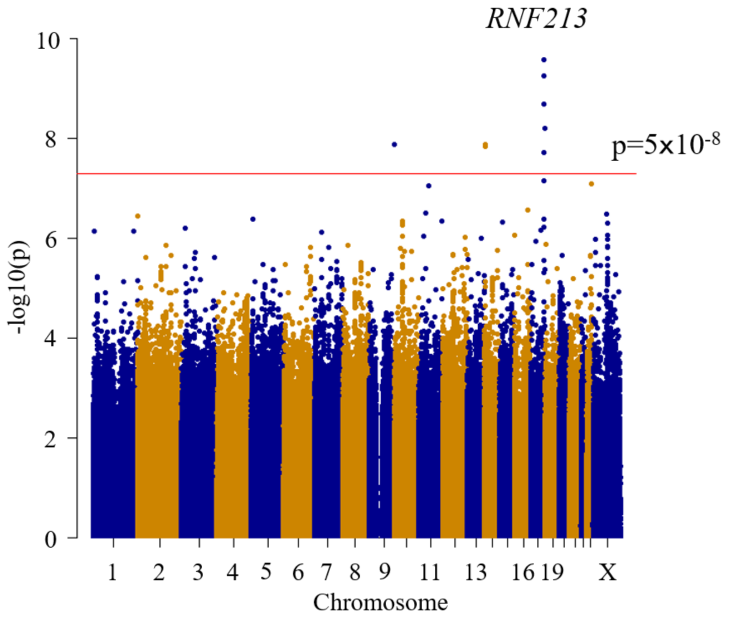

(b)

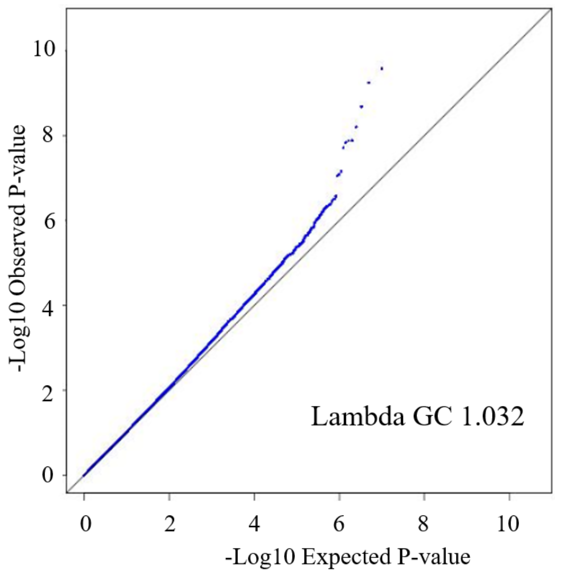

© 2024 Hikino K et al. *JAMA Cardiology*.

(a) Manhattan plot. Results are plotted as –log10 p-values on the y-axis by position on the chromosome (x-axis, NCBI build 37). The red line represents the genome-wide significance level (p-value = 5×10^{-8}). Gene names are shown next to the top loci.

(b) Q–Q plot showing observed versus expected P-values. GC, genomic control.

© 2024 Hikino K et al. *JAMA Cardiology*.

eFigure 4. Associations in the *RNF213* and VSA in the combined datasets.

This figure shows the results of the genetic associations in the *RNF213* region and vasospastic angina (VSA) using the first and second data sets.

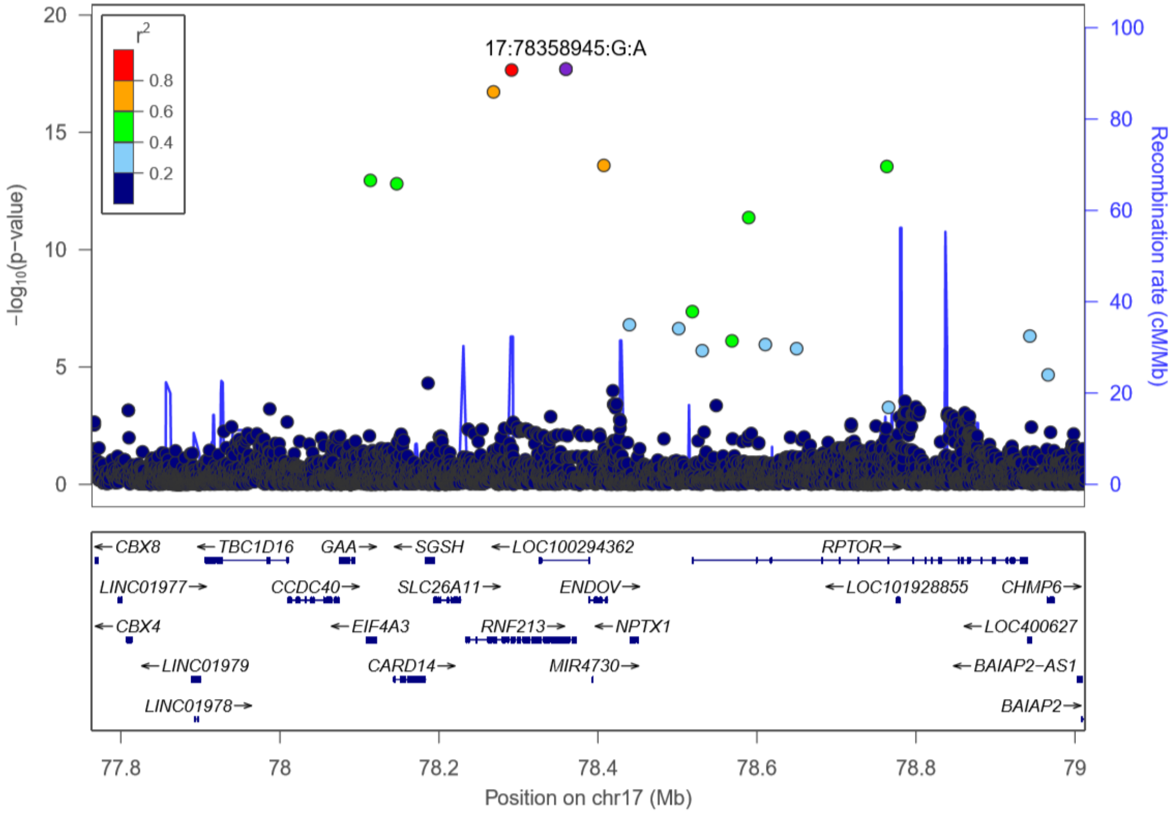

Colouring is based on linkage disequilibrium (LD) with the top hit in the VSA region.

© 2024 Hikino K et al. *JAMA Cardiology*.

eFigure 5. Effect sizes of the CAD-associated variants between VSA and CADs.

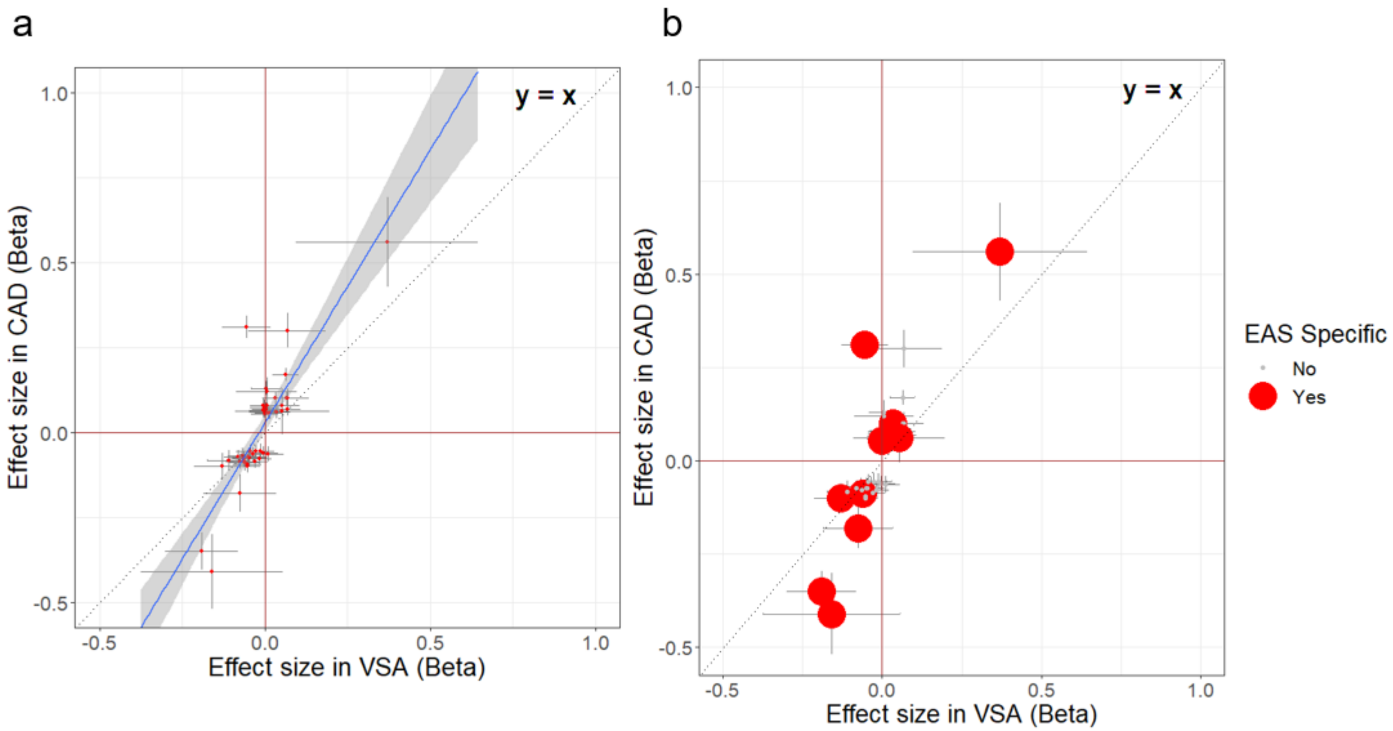

This figure displays the results of association analyses for the combined dataset. The X- and Y-axes show the beta coefficients of CAD-associated variants in VSA and non-VSA CAD, respectively. The bars crossing the red dots represent the 95% confidence intervals of the beta coefficients. The blue line shows the regression line with ties of the 95% confidence interval in grey. All CAD-associated variants (except rs112735431) are shown in a). EAS-specific variants are highlighted in (b).

© 2024 Hikino K et al. *JAMA Cardiology*.

CAD, coronary artery disease; VSA, Vasospastic angina; MI, myocardial infarction; EAS, East Asian.

© 2024 Hikino K et al. *JAMA Cardiology*.

eFigure 6. Effect sizes of rs112735431 between VSA and the CAD subtypes.

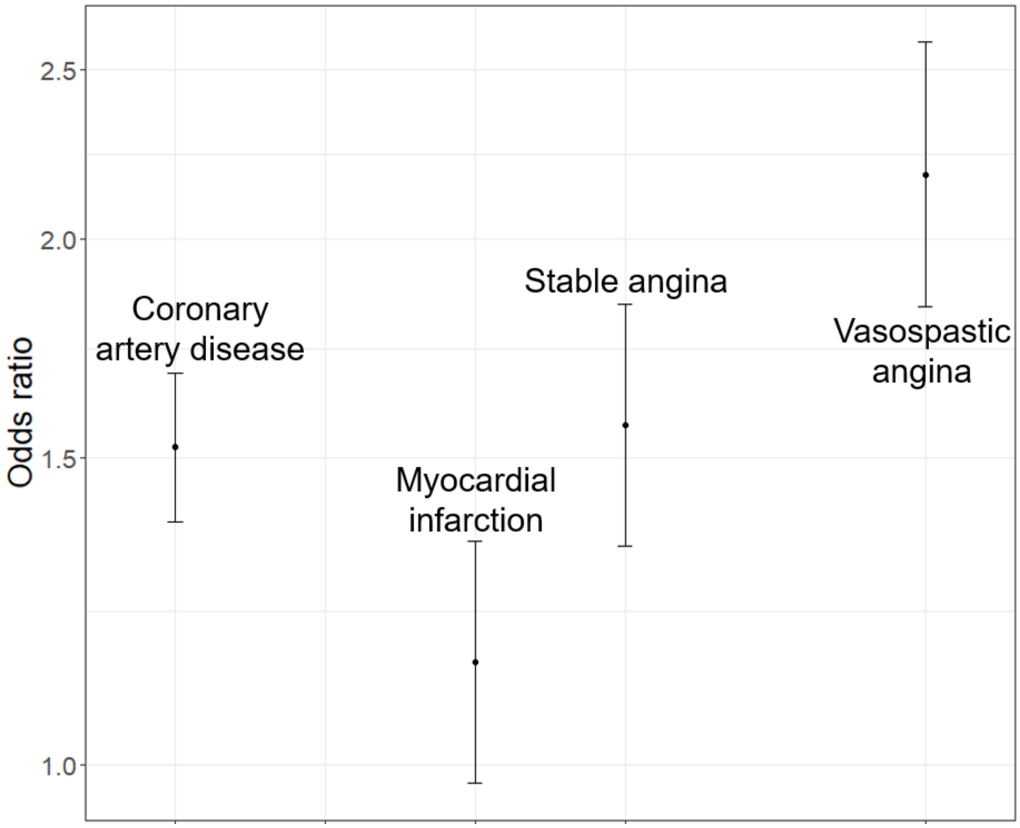

This figure shows the results of association analyses for the combined dataset. The X-axis shows the four phenotypes: non-VSA CAD, myocardial infarction, stable angina, and VSA. The Y-axis shows the odds ratio of rs112735431 in the four diseases. The bars crossing the black dots in the line graph represent the 95% confidence intervals of the odds ratios.

CAD, coronary artery disease; VSA, Vasospastic angina.

© 2024 Hikino K et al. *JAMA Cardiology*.

eFigure 7. Locus zoom plot of the results of the meta-analysis.

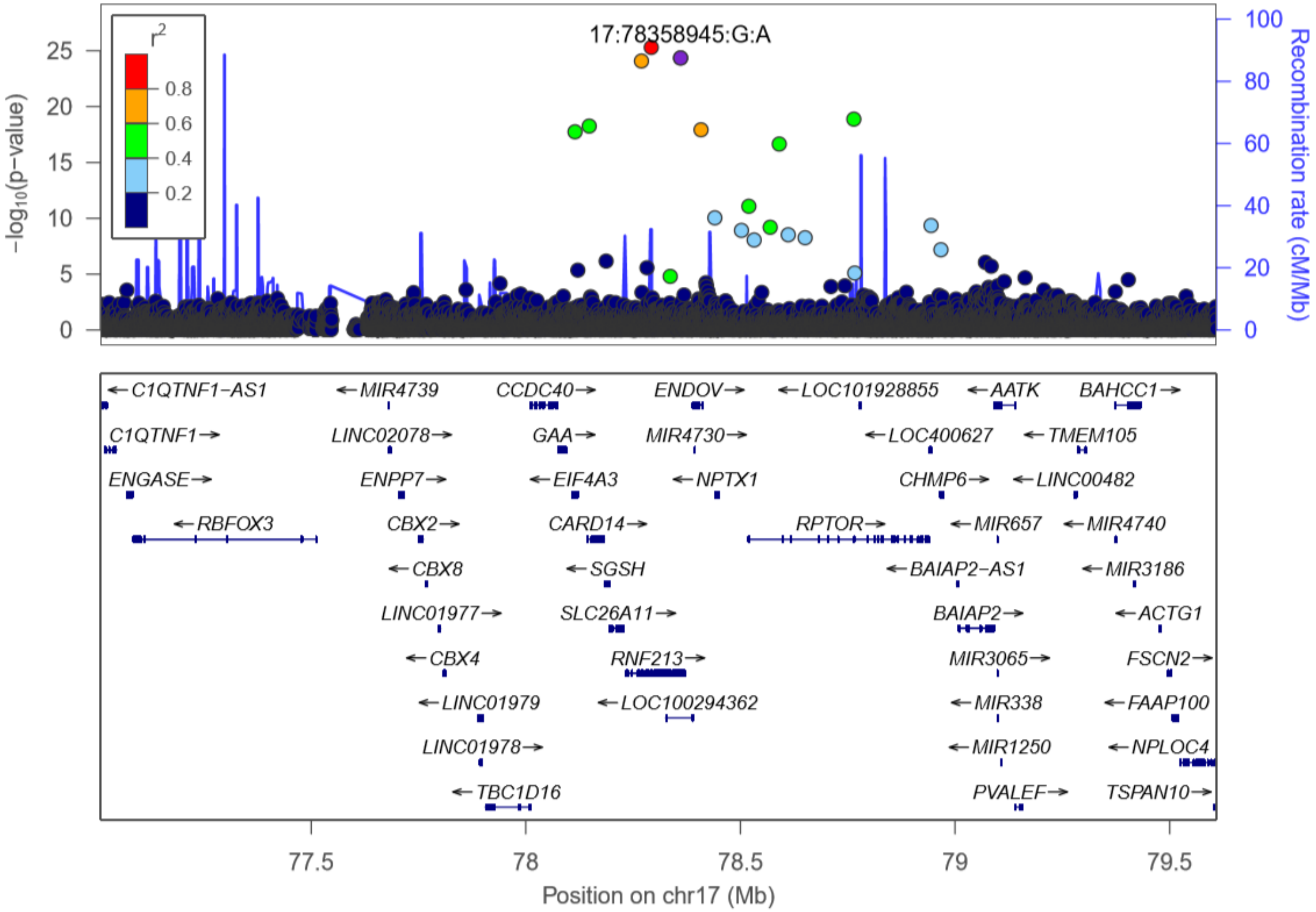

This plot shows the results of the meta-analysis in the *RNF213* region using all the datasets. Colouring was based on linkage disequilibrium (LD) with rs112735431.

© 2024 Hikino K et al. *JAMA Cardiology*.

eFigure 8. Locus zoom plot of the conditional analyses of rs112735431.

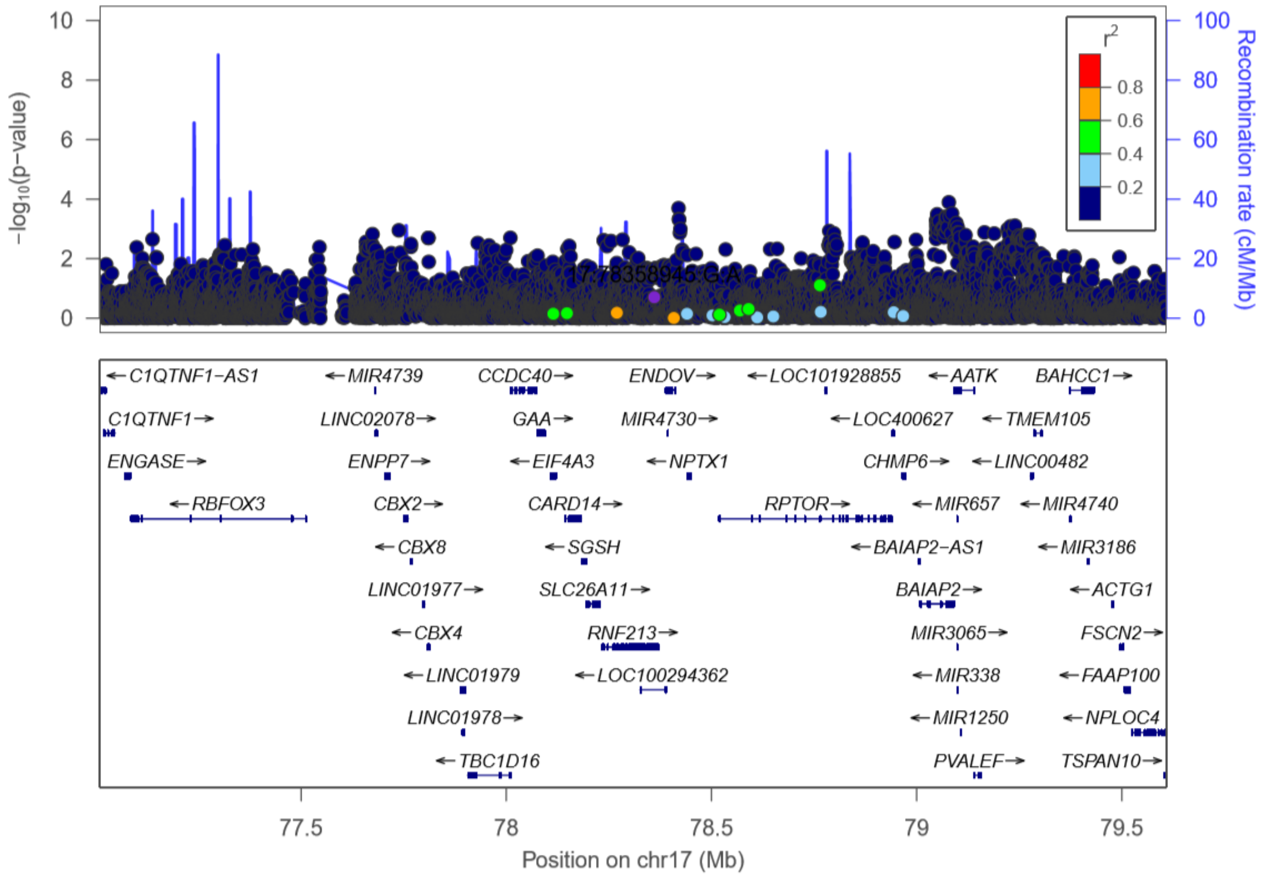

© 2024 Hikino K et al. *JAMA Cardiology*.

This figure plots the results of the conditional analyses of rs112735431 from the meta-analysis. Colouring was based on linkage disequilibrium (LD) with rs112735431.

© 2024 Hikino K et al. *JAMA Cardiology*.

eTable 1. Baseline characteristics of the patients included in this study.

Phenotype	N	Mean age (SD)	Men (%)	h/o smoking (%)	h/o alcohol consumption (%)	Hyperlipidemia (%)	Type 2 DM (%)	Hypertension (%)
**GWAS (1st and 2nd data sets)**								
Controls	143,964	61.8 (15)	n = 72,044 (50.0)	n = 66,873 (46.5)	n = 70,607 (49.0)	n = 42,135 (29.3)	n = 33,918 (23.6)	n = 32,285 (22.4)
Vasospastic angina	5,192	67.6 (10)	n = 3,345 (64.4)	n = 3,026 (58.3)	n = 2,875 (55.4)	n = 2,465 (47.5)	n = 1,302 (25.1)	n = 1,459 (28.1)
Stable angina pectoris	8,328	69.2 (9.5)	n = 5,899 (70.8)	n = 5,007 (60.1)	n = 4,219 (50.7)	n = 4,533 (54.4)	n = 3,073 (36.9)	n = 3,031 (36.4)
Myocardial infarction	11,028	69.2 (9.0)	n = 8,680 (78.7)	n = 7,571 (68.7)	n = 5,349 (48.5)	n = 6,263 (56.8)	n = 4,168 (37.8)	n = 3,262 (29.6)
**Additional data (3rd data set)**								
Controls	9,900	65.9	n = 5,383	n = 5,125	n = 5,155	n = 1,710	n = 141	n = 2,168

© 2024 Hikino K et al. *JAMA Cardiology*.

		(12.6)	(54.4)	(51.8)	(52.1)	(17.3)	(1.4)	(21.9)
Vasospastic angina	528	67.1<br>(11.4)	n = 328<br>(62.1)	n = 314<br>(59.5)	n = 327<br>(61.9)	n = 266<br>(50.4)	n = 32<br>(6.1)	n = 159<br>(30.1)

n, number; SD, standard deviation; h/o, history of DM, diabetes mellitus.

© 2024 Hikino K et al. *JAMA Cardiology*.

eTable 2. Associations with VSA identified in the first or second data sets.

Chr	variant ID (rs)	Gene	1st set AF. Cases/Ctrls	1st set OR (95% CI)	1st set P-value	2nd set AF. Cases/Ctrls	2nd set OR (95% CI)	2nd set P-value	Combined set (1st and 2nd) AF. Cases/Ctrls	Combined set OR (95% CI)	Combined set P-value
17	rs112735431	*RNF213*	0.016/0.0097	2.00 (1.62-2.47)	**1.2×10^{-10}**	0.020/0.0095	2.71 (1.99-3.69)	**2.7×10^{-10}**	0.017/0.0096	2.18 (1.83-2.59)	**$\underline{2.0\times10^{-18}}$**
17	rs111321460	*RNF213*	0.015/0.0091	2.09 (1.67-2.60)	**6.7×10^{-11}**	0.018/0.0091	2.79 (2.02-3.85)	**5.6×10^{-10}**	0.016/0.0091	2.26 (1.88-2.71)	**2.2×10^{-18}**
4	rs117812098	*GRXCR1*	0.063/0.051	1.34 (1.21-1.48)	**1.4×10^{-8}**	0.052/0.050	1.07 (0.89-1.29)	0.45	0.060/0.051	1.27 (1.16-1.39)	1.2×10^{-7}
1	rs55751537	*GRIK3*	0.024/0.015	1.56 (1.33-1.84)	**3.6×10^{-8}**	0.017/0.016	1.09 (0.80-1.47)	0.59	0.022/0.016	1.43 (1.24-1.65)	6.4×10^{-7}
14	rs201932150	*LOC124903296*	0.024/0.022	1.08 (0.92-1.26)	0.34	0.037/0.022	1.81 (1.47-2.22)	**1.3×10^{-8}**	0.027/0.022	1.27 (1.12-1.43)	0.00017
9	rs74979308	*OLFM1*	0.0089/0.0087	1.05	0.73	0.018/0.0088	2.37	**1.31×10^{-8}**	0.011/0.0087	1.38	0.0012

© 2024 Hikino K et al. *JAMA Cardiology*.

(0.81-1.36)	(1.76-3.19)	(1.14-1.68)

Allele frequencies of rs112735431 in 1000 Genomes: East Asian A=0.9980, T=0.0020; Europe A=1.0000, T=0.0000; African A=1.0000, T=0.0000; and in the Genome Aggregation Database (gnomAD): East Asian A=0.9973, T=0.0027; Europe A=1.0000, T=0.0000; African A=1.0000, T=0.0000.

Chr, chromosome; Ref, reference allele; Var, variant allele; AF.Cases, variant allele frequency in cases; AF.Controls, variant allele frequency in controls; OR, odds ratio; CI, confidence interval.

© 2024 Hikino K et al. *JAMA Cardiology*.

eTable 3. Comparison of effect sizes of CAD-associated variants among subgroups of CADs.

variant	rsID	Gene	CAD			VSA			Stable AP			MI		
			BETA	SE	P-value	BETA	SE	P-value	BETA	SE	P-value.NA	BETA	SE	P-value
1:55509585:C:T	rs151193009	*PCSK9*	-0.41	0.056	1.25×10^{-13}	-0.16	0.11	0.14	-0.40	0.095	2.83×10^{-5}	-0.53	0.089	1.98×10^{-9}
1:56884178:A:G	rs2184103	*LOC124904185*	0.062	0.010	1.54×10^{-10}	-0.0016	0.020	0.94	0.067	0.016	3.17×10^{-5}	0.077	0.014	6.52×10^{-8}
1:222822999:A:G	rs28709375	*MIA3*	0.10	0.010	5.86×10^{-23}	0.033	0.020	0.10	0.089	0.016	3.73×10^{-8}	0.13	0.014	3.67×10^{-20}
2:21242731:G:A	rs13306206	*APOB*	0.30	0.026	2.36×10^{-29}	0.068	0.060	0.26	0.30	0.043	2.53×10^{-12}	0.37	0.038	2.60×10^{-23}
2:85739984:A:G	rs9751370	*MAT2A*	0.058	0.010	2.05×10^{-9}	0.014	0.020	0.50	0.075	0.016	4.70×10^{-6}	0.057	0.014	7.40×10^{-5}
2:164927706:C:A	rs10930114	*FIGN*	-0.073	0.010	3.24×10^{-13}	-0.081	0.021	0.00011	-0.056	0.017	0.00076	-0.080	0.015	6.57×10^{-8}
2:230007146:C:T	rs62190384	*PID1*	-0.055	0.010	1.31×10^{-8}	-0.027	0.020	0.18	-0.061	0.016	0.00013	-0.064	0.014	5.72×10^{-6}
3:14886687:G:A	rs4395384	*FGD5*	-0.078	0.012	1.47×10^{-10}	-0.063	0.025	0.013	-0.085	0.020	2.86×10^{-5}	-0.086	0.018	1.64×10^{-6}
4:57765731:G:C	rs55762216	*REST*	0.057	0.010	5.27×10^{-9}	0.010	0.021	0.62	0.052	0.016	0.0017	0.090	0.014	4.39×10^{-10}
4:148407652:C:T	rs6841473	*EDNRA*	0.10	0.010	1.21×10^{-22}	0.066	0.022	0.0020	0.12	0.017	1.60×10^{-11}	0.092	0.015	1.44×10^{-9}
4:156442007:TCCAAGTTACTAG:T	rs56134367	*MTND1P22*	-0.055	0.011	2.02×10^{-7}	-0.013	0.022	0.56	-0.067	0.018	0.00015	-0.053	0.016	0.00069

© 2024 Hikino K et al. *JAMA Cardiology*.

5:4094165:G:T	rs10041378	*IRX1*	-0.064	0.011	2.03×10^{-8}	0.010	0.024	0.66	-0.065	0.019	0.00072	-0.086	0.017	4.87×10^{-7}
6:12903957:A:G	rs9349379	*PHACTR1*	0.13	0.011	7.05×10^{-32}	0.005	0.023	0.82	0.15	0.018	5.43×10^{-16}	0.17	0.016	8.95×10^{-26}
6:30989021:A:G	rs139141104	*MUC22*	0.062	0.034	0.070	0.053	0.073	0.47	0.070	0.057	0.22	0.070	0.050	0.17
6:44048051:C:T	rs149372871	*POLR1C*	-0.10	0.020	1.10×10^{-6}	-0.13	0.042	0.0019	-0.11	0.033	0.0017	-0.069	0.029	0.018
6:74415868:A:C	rs56171536	*CD109*	0.12	0.021	9.44×10^{-9}	0.006	0.047	0.90	0.10	0.036	0.0051	0.16	0.031	1.83×10^{-7}
6:134209837:T:C	rs2327429	*TCF21*	-0.10	0.010	5.87×10^{-24}	-0.052	0.020	0.0095	-0.11	0.016	9.74×10^{-13}	-0.10	0.014	2.14×10^{-13}
6:161001428:T:C	rs932631509	*LPA*	0.56	0.067	4.81×10^{-17}	0.37	0.14	0.0089	0.63	0.10	1.09×10^{-9}	0.65	0.093	2.21×10^{-12}
7:19052733:G:A	rs57301765	*TWIST1*	0.065	0.010	1.07×10^{-10}	0.0025	0.021	0.90	0.11	0.017	6.31×10^{-11}	0.052	0.015	0.00051
8:69431711:G:A	rs2380472	*C8orf34*	-0.082	0.015	4.50×10^{-8}	-0.11	0.032	0.00049	-0.10	0.025	0.00013	-0.059	0.022	0.0073
9:22092924:A:G	rs10811654	*CDKN2B-AS1*	0.17	0.010	2.72×10^{-68}	0.064	0.020	0.0014	0.18	0.016	9.23×10^{-28}	0.22	0.014	5.53×10^{-52}
9:107586238:C:A	rs35093463	*ABCA1*	0.054	0.010	5.09×10^{-8}	-0.00011	0.021	0.996	0.050	0.017	0.0029	0.078	0.015	1.21×10^{-7}
10:91002804:C:T	rs1412445	*LIPA*	0.060	0.011	9.51×10^{-8}	0.00067	0.024	0.98	0.044	0.019	0.020	0.093	0.017	2.19×10^{-8}
10:104829469:C:T	rs1926032	*CNNM2*	-0.087	0.011	1.47×10^{-14}	-0.061	0.024	0.0095	-0.084	0.019	1.02×10^{-5}	-0.092	0.017	3.81×10^{-8}
11:203235:A:G	rs73386640	*BET1L*	0.10	0.015	2.26×10^{-11}	0.069	0.031	0.026	0.11	0.024	6.28×10^{-6}	0.12	0.022	1.90×10^{-8}
11:65405600:G:T	rs2306363	*SIPA1*	-0.069	0.011	1.80×10^{-10}	-0.068	0.023	0.0030	-0.070	0.018	0.00012	-0.072	0.016	8.30×10^{-6}

© 2024 Hikino K et al. *JAMA Cardiology*.

11:103670449:T:C	rs61904693	*DDI1*	-0.087	0.010	1.35×10^{-17}	-0.076	0.021	0.00036	-0.082	0.017	1.47×10^{-6}	-0.10	0.015	4.65×10^{-12}
11:110244360:A:T	rs10488763	*LINC02732*	0.078	0.010	1.86×10^{-15}	-0.0042	0.021	0.84	0.10	0.016	2.42×10^{-9}	0.11	0.014	9.49×10^{-14}
12:10876573:C:A	rs2607903	*YBX3*	-0.063	0.010	1.43×10^{-10}	-0.035	0.021	0.090	-0.066	0.016	5.43×10^{-5}	-0.084	0.015	6.69×10^{-9}
12:54496219:A:G	rs1133773	*FLJ12825*	0.078	0.012	1.60×10^{-10}	0.053	0.026	0.039	0.060	0.020	0.0033	0.096	0.018	6.67×10^{-8}
12:90008959:A:G	rs2681472	*ATP2B1*	0.068	0.010	4.71×10^{-12}	0.0032	0.021	0.88	0.085	0.016	1.88×10^{-7}	0.091	0.014	3.55×10^{-10}
12:95521370:C:T	rs11107909	*FGD6*	-0.089	0.012	1.41×10^{-14}	-0.062	0.024	0.010	-0.077	0.020	7.98×10^{-5}	-0.10	0.017	9.39×10^{-9}
12:112168009:G:A	rs11066015	*ACAD10*	0.31	0.017	4.87×10^{-79}	-0.055	0.037	0.13	0.24	0.028	1.72×10^{-17}	0.55	0.024	3.74×10^{-114}
13:29042432:G:A	rs74412485	*FLT1*	-0.35	0.028	3.08×10^{-35}	-0.19	0.056	0.00094	-0.44	0.049	3.29×10^{-19}	-0.40	0.043	7.14×10^{-21}
13:110954237:A:G	rs4773140	*COL4A1*	0.074	0.011	7.51×10^{-12}	-0.0010	0.022	0.96	0.083	0.018	5.25×10^{-6}	0.12	0.016	6.82×10^{-13}
14:100111565:G:A	rs12893887	*HHIPL1*	-0.062	0.010	1.44×10^{-9}	-0.005	0.022	0.80	-0.034	0.017	0.052	-0.092	0.015	1.97×10^{-9}
15:79022616:G:A	rs8027011	LOC105370913	-0.095	0.010	3.99×10^{-21}	-0.053	0.021	0.012	-0.11	0.017	2.32×10^{-10}	-0.094	0.015	2.73×10^{-10}
15:89574418:T:C	rs2083458	*ABHD2*	-0.086	0.010	9.70×10^{-19}	-0.029	0.020	0.16	-0.11	0.016	1.16×10^{-11}	-0.10	0.014	5.46×10^{-12}
15:91428636:G:A	rs7177338	*FES*	-0.074	0.013	1.37×10^{-8}	-0.046	0.027	0.094	-0.10	0.022	1.58×10^{-6}	-0.087	0.019	5.10×10^{-6}
16:75306402:C:T	rs999675	*BCAR1*	0.068	0.010	1.74×10^{-12}	0.069	0.020	0.00065	0.077	0.016	2.19×10^{-6}	0.053	0.014	0.00018
17:59232365:T:C	rs11655024	*BCAS3*	-0.073	0.013	5.91×10^{-8}	-0.050	0.028	0.079	-0.062	0.023	0.0056	-0.10	0.020	1.42×10^{-7}

© 2024 Hikino K et al. *JAMA Cardiology*.

17:62399872:C:T	rs9902260	*PECAM1*	0.065	0.011	2.61×10^{-9}	-0.0040	0.022	0.86	0.045	0.018	0.012	0.11	0.016	2.94×10^{-11}
17:78358945:G:A	rs112735431	*RNF213*	0.42	0.050	7.52×10^{-17}	0.78	0.089	2.03×10^{-18}	0.45	0.081	3.48×10^{-8}	0.14	0.081	0.096
18:20009691:T:C	rs9951447	*CTAGE1*	0.061	0.010	1.44×10^{-9}	0.036	0.021	0.085	0.10	0.017	5.67×10^{-9}	0.052	0.015	0.00051
19:11253886:CA:C	rs34774090	*SPC24*	0.080	0.010	1.81×10^{-14}	0.006	0.022	0.79	0.072	0.017	3.59×10^{-5}	0.12	0.016	5.00×10^{-14}
19:16430677:G:T	rs10420373	*KLF2*	-0.056	0.010	1.72×10^{-8}	-0.044	0.021	0.036	-0.064	0.017	0.00012	-0.055	0.015	0.00018
19:41876468:T:C	rs4803459	*TMEM91*	-0.078	0.010	6.99×10^{-16}	-0.017	0.020	0.41	-0.092	0.016	1.72×10^{-8}	-0.10	0.014	8.12×10^{-12}
19:45425178:G:A	rs190712692	*APOC1*	-0.18	0.028	1.29×10^{-10}	-0.076	0.056	0.17	-0.22	0.047	2.62×10^{-6}	-0.22	0.042	2.40×10^{-7}

The variants were obtained from Koyama et al., Nat Genet 2020. variant IDs are chromosomes: position, reference allele, and alternate allele. The positions correspond to the hg19 or GRCh37 build. BETA was an alternative allele. CAD, coronary artery disease; VSA, vasospastic angina; AP, angina pectoris; SE, standard error.

© 2024 Hikino K et al. *JAMA Cardiology*.

eTable 4. Haplotype analysis of the variants at the *RNF213* locus.

Haplotype (rs111321460- rs112735431)	freq (case/control)	OR (95% CI)	P-value
C-A	0.0144/0.0066	2.20 (1.86-2.59)	3.5×10^{-18}
T-A	0.0017/0.0012	1.40 (0.83-2.23)	0.17
T-G	0.9840/0.9922	REF	-

Freq, haplotype frequency; OR, odds ratio; CI, confidence interval; P, p-value; REF, reference

© 2024 Hikino K et al. *JAMA Cardiology*.

eTable 5. Stratified analysis of the association between *RNF213* and vasospastic angina based on sex and age.

Chr	Position	variant ID (rs)	Gene	Ref/var	Group	AF.Cases	AF.Controls	BETA	SE	P-value
17	78358945	rs112735431	*RNF213*	G/A	Men	0.020	0.010	0.97	0.10	7.79×10^{-23}
					Women	0.014	0.010	0.63	0.15	2.59×10^{-5}
					<60 y.o.	0.022	0.010	1.12	0.16	1.09×10^{-12}
					60~<70 y.o.	0.018	0.010	0.86	0.14	1.08×10^{-9}
					70~ y.o.	0.015	0.0092	0.74	0.13	1.45×10^{-8}

Chr, chromosome; Ref, reference allele; Var, variant allele; AF.Cases, variant allele frequency in cases; AF.Ctrls, variant allele frequency in controls; SE, standard error; y.o., year-old.

© 2024 Hikino K et al. *JAMA Cardiology*.

eTable 6. Association between the *RNF213* locus and patients with vasospastic angina positive for drug-induced vasospasm.

Chr	Position	variant ID (rs)	Gene	Ref/var	AF.Cases	AF.Controls	OR (95%CI)	P-value
17	78358945	rs112735431	*RNF213*	G/A	0.021	0.0077	2.82 (1.49-5.36)	1.5×10^{-3}

Chr, chromosome; Ref, reference allele; Var, variant allele; AF.Cases, variant allele frequency in cases; AF.Ctrls, variant allele frequency in controls; OR, odds ratio; CI, confidence interval; y.o., year-old.

© 2024 Hikino K et al. *JAMA Cardiology*.

eTable 7. Non-shared effect direction of Moyamoya disease-associated variants between vasospastic angina and Moyamoya disease.

Marker	Gene	Allele1/2	A1 Freq in EA/EUR	A1 Effect (VSA)	StdErr	P-value	Direction concordance
1:11862214:T:C	*MTHFR*	t/c	0.59/0.81	0.028	0.025	0.26	+
3:150178939:C:T	*TSC22D2*	t/c	0.31/0.11	0.0045	0.020	0.82	+
7:19049388:G:A	*TWIST1*	a/g	0.36/0.17	-0.0033	0.021	0.88	-
7:28179396:C:T	*JAZF1*	t/c	0.50/0.61	-0.019	0.020	0.35	-
11:61534010:C:T	*MYRF*	t/c	0.58/0.31	0.0093	0.022	0.67	+
11:9842491:C:T	*SBF2*	t/c	0.32/0.88	0.020	0.021	0.35	+
12:57533690:C:A	*LRP1*	a/c	0.24/0.32	-0.0070	0.026	0.79	-
18:20104982:T:C	*LOC124904265*	t/c	0.25/0.16	-0.015	0.024	0.53	+
20:12732879:C:T	*SPTLC3*	t/c	0.037/0.34	-0.028	0.045	0.53	-
22:30601243:G:A	*HORMAD2*	a/g	0.19/0.40	-0.0039	0.027	0.89	-

Direction concordance: shared direction of allele effects between Moyamoya disease and VSA (+ indicates shared and non-shared directions of association between Moyamoya disease and VSA). variant IDs are chromosomes: position, reference allele, and alternate allele.

© 2024 Hikino K et al. *JAMA Cardiology*.

eTable 8. Overview of the included datasets in each analysis

Analysis	Datasets				
	1st	2nd	Combined (1st, 2nd)	Additional (3rd)	All datasets (1st, 2nd, 3rd)
Genome-wide association study	○	○	○	○	
Heritability estimation			○		
Comparison of effect sizes between VSA and non-VSA CADs			○		
Evaluation of genetic correlations			○		
Haplotype analysis					○
Evaluation of effects size of rs112735431 in heterozygote or homozygote individuals					○
Stratified analyses based on sex or registered age					○
Correlation analysis between Moyamoya disease susceptibility variants			○		
Survival analysis for patients in the BBJ			○		

© 2024 Hikino K et al. *JAMA Cardiology*.

eReferences

1. Nagai A, Hirata M, Kamatani Y, et al. Overview of the BioBank Japan Project: Study design and profile. *J Epidemiol.* 2017;27(3S):S2-S8.
2. Hikino K, Koido M, Otomo N, et al. Genome-wide association study of colorectal polyps identified highly overlapping polygenic architecture with colorectal cancer. *J Hum Genet.* 2021.
3. Hikino K, Koido M, Tomizuka K, et al. Susceptibility loci and polygenic architecture highlight population specific and common genetic features in inguinal hernias: genetics in inguinal hernias. *EBioMedicine.* 2021;70:103532.
4. Purcell S, Neale B, Todd-Brown K, et al. PLINK: a tool set for whole-genome association and population-based linkage analyses. *Am J Hum Genet.* 2007;81(3):559-575.
5. Genomes Project C, Abecasis GR, Altshuler D, et al. A map of human genome variation from population-scale sequencing. *Nature.* 2010;467(7319):1061-1073.
6. International HapMap C, Altshuler DM, Gibbs RA, et al. Integrating common and rare genetic variation in diverse human populations. *Nature.* 2010;467(7311):52-58.
7. Manichaikul A, Mychaleckyj JC, Rich SS, Daly K, Sale M, Chen WM. Robust relationship inference in genome-wide association studies. *Bioinformatics.* 2010;26(22):2867-2873.
8. Pruim RJ, Welch RP, Sanna S, et al. LocusZoom: regional visualization of genome-wide association scan results. *Bioinformatics.* 2010;26(18):2336-2337.
9. Yang J, Ferreira T, Morris AP, et al. Conditional and joint multiple-SNP analysis of GWAS summary statistics identifies additional variants influencing complex traits. *Nat Genet.* 2012;44(4):369-375, S361-363.
10. Khera AV, Chaffin M, Aragam KG, et al. Genome-wide polygenic scores for common diseases identify individuals with risk equivalent to monogenic mutations. *Nat Genet.* 2018;50(9):1219-1224.
11. Bulik-Sullivan BK, Loh PR, Finucane HK, et al. LD Score regression distinguishes confounding from polygenicity in genome-wide association studies. *Nat Genet.* 2015;47(3):291-295.
12. Koyama S, Ito K, Terao C, et al. Population-specific and trans-ancestry genome-wide analyses identify distinct and shared genetic risk loci for coronary artery disease. *Nat Genet.* 2020;52(11):1169-1177.

© 2024 Hikino K et al. *JAMA Cardiology*.

13. Ogawa H, Suefuji H, Soejima H, et al. Increased blood vascular endothelial growth factor levels in patients with acute myocardial infarction. *Cardiology.* 2000;93(1-2):93-99.
14. Group JCSJW. Guidelines for diagnosis and treatment of patients with vasospastic angina (Coronary Spastic Angina) (JCS 2013). *Circ J.* 2014;78(11):2779-2801.
15. Wang K, Li M, Hakonarson H. ANNOVAR: functional annotation of genetic variants from high-throughput sequencing data. *Nucleic Acids Res.* 2010;38(16):e164.
16. Kanai M, Akiyama M, Takahashi A, et al. Genetic analysis of quantitative traits in the Japanese population links cell types to complex human diseases. *Nat Genet.* 2018;50(3):390-400.
17. Willer CJ, Li Y, Abecasis GR. METAL: fast and efficient meta-analysis of genomewide association scans. *Bioinformatics.* 2010;26(17):2190-2191.
18. Lenz TL, Deutsch AJ, Han B, et al. Widespread non-additive and interaction effects within HLA loci modulate the risk of autoimmune diseases. *Nat Genet.* 2015;47(9):1085-1090.
19. Consortium EP, Moore JE, Purcaro MJ, et al. Expanded encyclopaedias of DNA elements in the human and mouse genomes. *Nature.* 2020;583(7818):699-710.
20. Boyle AP, Hong EL, Hariharan M, et al. Annotation of functional variation in personal genomes using RegulomeDB. *Genome Res.* 2012;22(9):1790-1797.
21. Megill. C, Martin. B, Weaver. C, et al. cellxgene: a performant, scalable exploration platform for high dimensional sparse matrices. *bioRxiv.* 2021.
22. Liu X, Jian X, Boerwinkle E. dbNSFP: a lightweight database of human nonsynonymous SNPs and their functional predictions. *Hum Mutat.* 2011;32(8):894-899.
23. Liu X, Li C, Mou C, Dong Y, Tu Y. dbNSFP v4: a comprehensive database of transcript-specific functional predictions and annotations for human nonsynonymous and splice-site SNVs. *Genome Med.* 2020;12(1):103.
24. Duan L, Wei L, Tian Y, et al. Novel Susceptibility Loci for Moyamoya Disease Revealed by a Genome-Wide Association Study. *Stroke.* 2018;49(1):11-18.
25. Terao C, Suzuki A, Momozawa Y, et al. Chromosomal alterations among age-related haematopoietic clones in Japan. *Nature.* 2020;584(7819):130-135.
26. Kim JS. Moyamoya Disease: Epidemiology, Clinical Features, and Diagnosis. *J Stroke.* 2016;18(1):2-11.

© 2024 Hikino K et al. *JAMA Cardiology*.

27. Larson AS, Savastano L, Klaas J, Lanzino G. Cardiac manifestations in a western moyamoya disease population: a single-center descriptive study and review. *Neurosurg Rev.* 2021;44(3):1429-1436.
28. Group JCSJW. Guidelines for diagnosis and treatment of patients with vasospastic angina (coronary spastic angina) (JCS 2008): digest version. *Circ J.* 2010;74(8):1745-1762.
29. Mineharu Y, Miyamoto S. RNF213 and GUCY1A3 in Moyamoya Disease: Key Regulators of Metabolism, Inflammation, and Vascular Stability. *Front Neurol.* 2021;12:687088.

© 2024 Hikino K et al. *JAMA Cardiology*.
