# Supplementary material for: RNF213 Variants, Vasospastic Angina, and Risk of Fatal Myocardial Infarction
Source: JAMA Cardiol. 2024 Jun 18;9(8):723–31. doi: 10.1001/jamacardio.2024.1483 (PMC11195602; doi:10.1001/jamacardio.2024.1483)
Supplement: Supplement 2. — Nonauthor Collaborators. The Biobank Japan Project. [file jamacardiol-e241483-s002.pdf]

Supplemental Online Content: Nonauthor Collaborators
*First name, last name, and suffix (if applicable) are required and will appear in PubMed.

*Group Name(s): The Biobank Japan Project							
***First Name and Middle Initial(s)**	***Last Name**	***Suffix (eg, Jr, III)**	Academic Degrees	Institution	Location (city, state/province, country)	Role or Contribution, eg, chair, principal investigator	Group (if more than 1 Group listed in the byline) and/or Subgroup (eg, Steering Committee)
Yuji	Yamanashi		PhD	The University of Tokyo	Tokyo, Japan	Member	BioBank Japan Project Consortium
Yoichi	Furukawa		PhD	The University of Tokyo	Tokyo, Japan	Member	BioBank Japan Project Consortium
Yoshinori	Murakami		PhD	The University of Tokyo	Tokyo, Japan	Member	BioBank Japan Project Consortium
Kaori	Muto		PhD	The University of Tokyo	Tokyo, Japan	Member	BioBank Japan Project Consortium
Akiko	Nagai		PhD	The University of Tokyo	Tokyo, Japan	Member	BioBank Japan Project Consortium
Wataru	Obara		PhD	Iwate Medical University	Iwate, Japan	Member	BioBank Japan Project Consortium
Ken	Yamaji		PhD	Juntendo University Graduate School of Medicine	Tokyo, Japan	Member	BioBank Japan Project Consortium
Kazuhisa	Takahashi		PhD	Juntendo University Graduate School of Medicine	Tokyo, Japan	Member	BioBank Japan Project Consortium
Satoshi	Asai		PhD	Nihon University	Tokyo, Japan	Member	BioBank Japan Project Consortium
Yasuo	Takahashi		PhD	Nihon University	Tokyo, Japan	Member	BioBank Japan Project Consortium
Takao	Suzuki		PhD	Tokushukai Group	Tokyo, Japan	Member	BioBank Japan Project Consortium
Nobuaki	Sinozaki		PhD	Tokushukai Group	Tokyo, Japan	Member	BioBank Japan Project Consortium
Hiroki	Yamaguchi		PhD	Nippon Medical School	Tokyo, Japan	Member	BioBank Japan Project Consortium
Shiro	Minami		PhD	Nippon Medical School	Tokyo, Japan	Member	BioBank Japan Project Consortium
Shigeo	Murayama		PhD	Tokyo Metropolitan Geriatric Hospital and Institute of Gerontology	Tokyo, Japan	Member	BioBank Japan Project Consortium
Kozo	Yoshimori		PhD	Fukujuji Hospital	Tokyo, Japan	Member	BioBank Japan Project Consortium
Satoshi	Nagayama		PhD	The Cancer Institute Hospital of the Japanese Foundation for Cancer Research	Tokyo, Japan	Member	BioBank Japan Project Consortium
Daisuke	Obata		PhD	Shiga University of Medical Science	Shiga, Japan	Member	BioBank Japan Project Consortium

Supplemental Online Content: Nonauthor Collaborators
*First name, last name, and suffix (if applicable) are required and will appear in PubMed.

***First Name and Middle Initial(s)**	***Last Name**	***Suffix (eg, Jr, III)**	Academic Degrees	Institution	Location (city, state/province, country)	Role or Contribution, eg, chair, principal investigator	Group (if more than 1 Group listed in the byline) and/or Subgroup (eg, Steering Committee)
Masahiko	Higashiyama		PhD	Osaka International Cancer Institute	Osaka, Japan	Member	BioBank Japan Project Consortium
Akihide	Matsumoto		PhD	IIZUKA HOSPITAL	Fukuoka, Japan	Member	BioBank Japan Project Consortium
Yukihiro	Koretsune		PhD	National Hospital Organization Osaka National Hospital	Osaka, Japan	Member	BioBank Japan Project Consortium
