# Supplementary material for: RNF213 Variants, Vasospastic Angina, and Risk of Fatal Myocardial Infarction
Source: JAMA Cardiol. 2024 Jun 18;9(8):723–31. doi: 10.1001/jamacardio.2024.1483 (PMC11195602; doi:10.1001/jamacardio.2024.1483)
Supplement: Supplement 3. — Data Sharing Statement. [file jamacardiol-e241483-s003.pdf]

Data Sharing Statement

Hikino. RNF213 Variants, Vasospastic Angina, and Risk of Fatal Myocardial Infarction. *JAMA Cardiol*. Published June 18, 2024. doi:10.1001/jamacardio.2024.1483

Data
Data available: Yes
Data types: Other (please specify)
Additional Information: summary statistics
How to access data: The full GWAS results can be accessed through the website of the Japanese ENcyclopedia of GEnetic Associations by Riken (JENGER, http://jenger.riken.jp/en/).
When available: With publication

Supporting Documents
Document types: None

Additional Information
Who can access the data: Researchers whose proposed use of the data has been approved
Types of analyses: For any purpose
Mechanisms of data availability: With investigator support
